# Preventing trogocytosis by cathepsin B inhibition augments CAR T-cell function

**DOI:** 10.1038/s41392-026-02654-z

**Published:** 2026-04-22

**Authors:** Kenneth A. Dietze, Kiet Nguyen, Aashli Pathni, Frank Fazekas, Wenxiang Sun, Ethan Rosati, Jillian M. Baker, Maday Galeana Figueroa, Etse Gebru, Daniel Yamoah, Rediet Mulatu, Alexander Wang, Aaron P. Rapoport, David H. Lum, Xiaoxuan Fan, Sabarinath V. Radhakrishnan, Djordje Atanackovic, Arpita Upadhyaya, Tim Luetkens

**Affiliations:** 1 Department of Microbiology and Immunology, University of Maryland School of Medicine, Baltimore, MD USA; 2 Biophysics Graduate Program, University of Maryland, College Park, MD USA; 3 Biological Sciences Graduate Program, University of Maryland, College Park, MD USA; 4 Preclinical Research Resource, Huntsman Cancer Institute, Salt Lake City, UT USA; 5 Division of Hematology and Oncology, Medical College of Wisconsin, Milwaukee, WI USA; 6 Department of Medicine and Transplant/Cell Therapy Program, University of Maryland School of Medicine and Marlene and Stewart Greenebaum, Baltimore, USA; 7 Institute for Physical Science and Technology, University of Maryland, College Park, MD USA; 8 Department of Physics, University of Maryland, College Park, MD USA

**Keywords:** Biotechnology, Oncology, Tumour immunology

## Abstract

Chimeric antigen receptor (CAR) T-cell therapy has shown remarkable efficacy in cancer treatment. Nevertheless, most patients receiving CAR T cells relapse within 5 years of treatment. CAR-mediated trogocytosis (CMT) is a potential tumor escape mechanism in which cell surface proteins transfer from tumor cells to CAR T cells. CMT results in the emergence of antigen-negative tumor cells, which can evade future CAR detection, and antigen-positive CAR T cells, which have been suggested to cause CAR T-cell fratricide and exhaustion. Whether CMT indeed causes CAR T-cell dysfunction and the molecular mechanisms conferring CMT remain unknown. Using a selective degrader of trogocytosed antigen in CAR T cells, we show that the presence of trogocytosed antigen on the CAR T-cell surface directly causes CAR T-cell fratricide and exhaustion. By performing small molecule screening using a custom high-throughput CMT screening assay, we found that the cysteine protease cathepsin B is essential for CMT and that inhibition of cathepsin B is sufficient to prevent CAR T-cell fratricide and exhaustion, leading to improved long-term in vitro and in vivo CAR T-cell persistence and in vitro antitumor activity. Our data demonstrate that it is feasible to separate CMT from cytotoxic activity, that CAR T-cell persistence, a key factor associated with clinical CAR T-cell efficacy, is directly linked to cathepsin B activity in CAR T cells, and that it is possible to improve CAR T-cell function through selective inhibition of CMT.

## Introduction

Chimeric antigen receptors (CAR) are genetically engineered proteins combining antigen-binding domains with immune cell activation domains.^1–3^ T cells and natural killer (NK) cells can be engineered to express CARs to recognize tumor-associated antigens with high specificity and have been shown to be clinically effective at targeting several hematological malignancies.^1,4–7^ Currently, seven CAR T-cell approaches targeting CD19 or B-cell maturation antigen (BCMA) have been approved in the US for the treatment of hematological cancers.^8,9^ While CAR T-cell therapy has greatly improved clinical outcomes for patients, many patients still relapse within five years of treatment.^10–15^ Several factors have been implicated in CAR T-cell-associated relapse, including increased CAR T-cell exhaustion,^16^ reduced CAR T-cell expansion^17^ and persistence,^18,19^ and tumor antigen escape.^18,20,21^ It has previously been shown that CAR T cells and CAR NK cells rapidly transfer the targeted antigen from tumor cells to their own cell surface in a process called trogocytosis.^22–24^ Trogocytosis was initially observed at the immune synapse between conventional T cells and antigen presenting cells (APCs), where TCR internalization following activation resulted in the transfer of MHC to recipient T cells.^25,26^ CAR-mediated trogocytosis (CMT) occurs in both hematological and solid tumors and is correlated with increased expression of apoptotic and exhaustion markers on CAR T cells, as well as reduced persistence and expansion.^22–24^ In addition, it has been shown that T cells engineered to express the tumor antigen CD19 are efficiently killed by other CD19 CAR T cells (“fratricide”).^22^ CMT is also implicated in a reduction in target antigen on the tumor cell surface, leading to antigen-negative or antigen-low variants that could escape CAR T-cell detection.^23^ Reductions in CAR T-cell affinity have previously been shown to reduce CMT,^23^ but this could lead to an increased antigen threshold,^27^ preventing the detection of antigen-low tumor cells.

Understanding the mechanistic basis of CMT, determining its impact on CAR T-cell function, and identifying strategies to minimize its deleterious effects may lead to the development of more potent CAR T-cell therapies and increased overall efficacy of CAR T-cell therapies across indications.

The mechanism linking CMT to exhaustion, fratricide, and reduced CAR T-cell persistence has thus far been elusive due to the lack of specific inhibitors of trogocytosis. In the absence of data supporting this link, it is possible that CMT could itself be a secondary effect, and identifying strategies to mitigate CMT may not directly enhance CAR T-cell function. In addition, the molecular drivers of CMT remain poorly understood, complicating the development of targeted strategies to limit CMT. Here, we develop novel tools to answer the question of whether CMT causes CAR T-cell dysfunction and investigate processes that may contribute to CMT using small-molecule inhibitors and transgenic approaches.

We here demonstrate for the first time that antigen transferred to CAR T-cells via CMT directly causes T-cell dysfunction. In addition, we show that a novel luciferase-complementation assay is an effective approach to quantify CMT kinetics and use this assay to identify the cysteine protease cathepsin B (CTSB) as a key driver of CMT. We show that inhibition of CTSB efficiently prevents CAR T-cell fratricide and exhaustion, and confers improved long-term in vitro and in vivo CAR T-cell persistence. This work demonstrates that specific inhibition of CMT is desirable and feasible, and that targeting CMT by CTSB inhibition decreases CAR T-cell dysfunction and may increase CAR T-cell potency.

## Results

### CAR T cells acquire target antigen via trogocytosis

**Fig. 1:**
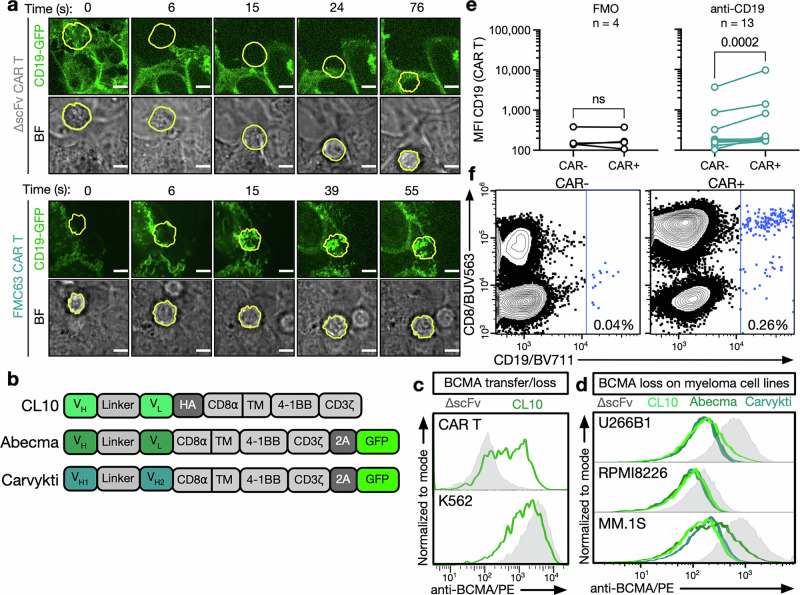
CD19 and BCMA CAR T cells rapidly acquire target antigens via trogocytosis. **a** Visualization of CMT in brightfield (BF) and confocal images of ∆scFv or FMC63 CAR T cells. CAR T cells were cultured with plate-bound 293 T cells expressing a CD19-GFP fusion protein. CD19 is shown in green. Individual CAR T cells are circled in yellow and tracked over time. Scale bar represents 5 µm. Data are representative of cells from two independent experiments. **b** Schema of BCMA-targeting CAR constructs. The same PBMC donor was used to produce Abecma and Carvykti CAR T cells; due to availability, a separate donor was used to produce CL10 CAR T cells. **c** BCMA transfer to CAR T cells (top) and BCMA loss in BCMA-GFP-expressing K562 cells (bottom) as determined by flow cytometry. **d** BCMA loss in MM.1S, RPMI8226, and U266B1 cells cocultured with CL10,^82^ Abecma, and Carvykti^79^ CAR T cells as determined by flow cytometry. **c**, **d** Data are representative of three independent experiments. **e** Amount of CD19 on CAR T cells (CAR^+^) or non-CAR T cells (CAR^−^) in PBMCs isolated from patients 7–28 days after receiving CD19 CAR T-cell therapy using full-spectrum flow cytometry. Data indicate values from individual patient samples (FMO *n* = 4; anti-CD19 *n* = 13). Statistical significance was determined by the Wilcoxon matched-pairs signed-rank test. Fluorescence-minus-one (FMO) refers to staining with a full flow cytometry panel except anti-CD19. **f** Representative flow cytometry data from one patient depicting CD19 levels on CAR^−^ and CAR^+^ T cells. CAR was stained using an anti-idiotype antibody

CMT is the extraction of the targeted tumor antigen from the tumor cell surface and its incorporation into the CAR T-cell plasma membrane^22–24,28^ (Supplementary Fig. S1a). Ex vivo, CMT occurs across cancer types^22,23^ and target antigens, including CD19,^22–24^ CD22,^22^ mesothelin,^22^ and BCMA.^28^ We found robust transfer of CD19 to CD19-targeting CAR T cells (clone: FMC63, Supplementary Fig. S1b) as well as CD19 loss on tumor cells (Supplementary Fig. S1c), which can be observed minutes after CAR T cells contact target cells (Fig. 1a and Supplementary Fig. S1d). Similarly, we confirmed the transfer of BCMA, the target of two clinically approved CAR T-cell approaches for the treatment of multiple myeloma^4,5,29,30^ (Fig. 1b), to BCMA CAR T cells (Fig. 1c) and the loss of BCMA from tumor cells (Fig. 1d) independent of the BCMA binding domain. When assessing CMT in solid tumor models in vitro, we observed significant transfer of target antigen to CAR T cells targeting LINGO1^31^ (Supplementary Fig. S1e), GD2 (Supplementary Fig. S1f), and FolRα (Supplementary Fig. S1g). In the clinical setting, CMT has thus far only been shown to occur in B-cell lymphoma patients treated with CAR NK cells.^24^ By analyzing peripheral blood mononuclear cells (PBMCs) from patients who had recently received CD19 CAR T cells, we demonstrated that CMT also occurs in some patients treated with CAR-expressing T cells (Fig. 1e, f and Supplementary Fig. S1h). Further analyzing a sample from a patient with a high number of CD19^+^ CAR T cells, we found that the presence of CD19 on CAR T cells was associated with increased levels of the exhaustion markers PD-1 and TIM-3 (Supplementary Fig. S1i).

### CMT directly causes CAR T-cell dysfunction

**Fig. 2:**
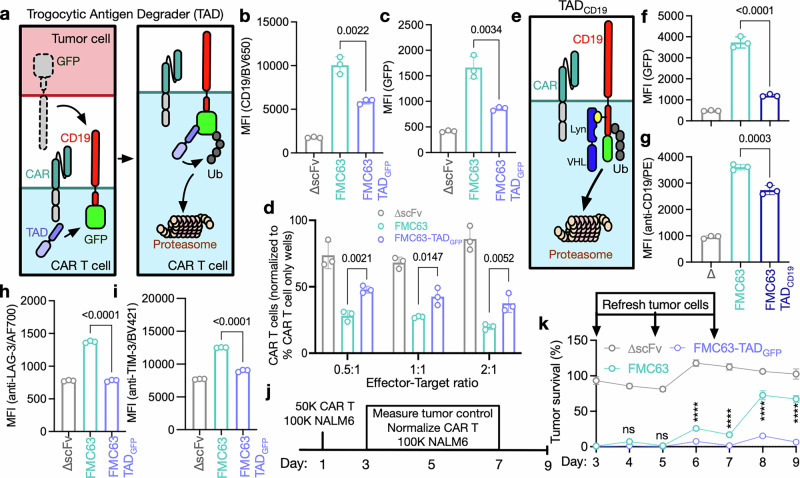
CMT directly causes CAR T-cell dysfunction. **a** Schema of the TAD approach. **b** CD19 and **c** GFP expression on FMC63 CAR T cells expressing TAD_GFP_ or T cells expressing a CAR lacking a binding domain (∆scFv) following a 2-h coculture with CD19-GFP-expressing NALM6 cells as determined by flow cytometry. Data indicate the mean ± S.D. from three technical replicates. **d** Quantification of CAR T cells after a 24-h coculture with CD19-GFP-expressing NALM6 cells using flow cytometry. Values are normalized to wells containing only CAR T cells. **b**–**d** Data indicate the mean ± S.D. from three technical replicates. Data are representative of three independent experiments. **e** Schema of a fully human-derived TAD system (TAD_CD19_). Lyn = SH2 domain of lyn kinase; VHL von Hippel‒Lindau protein, Ub ubiquitin. **f** GFP and **g** CD19 expression on FMC63 CAR T cells expressing TAD_CD19_ or ∆scFv CAR T cells after a 2-h coculture with CD19-GFP-expressing NALM6 cells at a 0.5:1 effector-target ratio. Data indicate the mean ± S.D. from three technical replicates. The data are representative of two independent experiments. **h** LAG-3 and **i** TIM-3 expression on CAR T cells expressing TAD_GFP_ as measured by MFI after a 24-h coculture with CD19-GFP-expressing NALM6 cells using full-spectrum flow cytometry. Data indicate the mean ± S.D. from three technical replicates. Data are representative of three independent experiments. **b**–**d**, **f**–**i** Statistical significance was determined by one-way ANOVA. **j** Schema of the in vitro serial coculture experiment to determine long-term CAR T-cell expansion and antitumor activity. CAR T cells were normalized, and fresh tumor cells were added on days 3, 5, and 7. **k** Survival of CD19-GFP-expressing NALM6 cells expressing firefly luciferase (Fluc) at a 0.5:1 effector-target ratio using a luminescence-based cytotoxicity assay. Data indicate the mean ± S.D. from three technical replicates. Tumor survival was normalized to untreated tumor cells. The data are representative of two independent experiments. Statistical significance comparing FMC63 and FMC63-TAD_GFP_ was determined by two-way ANOVA

The presence of trogocytosed antigen on CAR T cells is correlated with increased exhaustion and reduced viability^,^^22–24^, but it remains unknown whether CMT directly causes this T-cell dysfunction. To answer this question, we developed an approach for the targeted degradation of trogocytosed CD19 fused to GFP (CD19-GFP) in CAR T cells (Fig. 2a). To validate this approach, we first expressed CD19-GFP as well as a trogocytosed antigen degrader (TAD), the E3-targeting domain Nslmb^32,33^ fused to a GFP-binding protein (TAD_GFP_), in 293 T cells. TAD overexpression resulted in a significant reduction in total CD19, with multiple CD19 bands likely reflecting differential glycosylation^34,35^ (Supplementary Fig. S2a, b). We next generated CAR T cells expressing TAD_GFP_ and did not observe an effect of TAD_GFP_ on CAR T-cell expansion (Supplementary Fig. S2c) or short-term antitumor activity (Supplementary Fig. S1d).

When cocultured for 2 h with K562 cells expressing CD19-GFP, conventional FMC63 CAR T cells showed increased levels of CD19 (Fig. 2b and Supplementary Fig. S2e) and GFP (Fig. 2c and Supplementary Fig. S2f), indicative of CMT, as expected. However, FMC63 CAR T cells also expressing TAD_GFP_ showed an approximately 50% reduction in both CD19 and GFP after coculture. Using the TAD system, we next investigated the effect of CMT on CAR T-cell fratricide. We found that TAD_GFP_ expression did not alter the proliferation of CAR T cells when cocultured with CD19^+^ tumor cells (Supplementary Fig. S2g). However, TAD_GFP_ expression resulted in a twofold increase in CAR T-cell numbers after coculture (Fig. 2d), indicating that CMT directly causes CAR T-cell death, likely due to fratricide. Targeted degradation of the trogocytosed antigen was also validated using a fully human TAD (TAD_CD19_) construct directly targeting CD19. TAD_CD19_ recognizes CD19 by using the Lyn kinase SH2 domain (Fig. 2e), which has been shown to bind the intracellular domain of CD19 with high affinity.^36^ Coexpression of TAD_CD19_ in FMC63 CAR T cells also resulted in reduced CMT (Fig. 2f/g). The reduction in CD19-GFP on the CAR T-cell surface by TAD_CD19_ was more pronounced when measuring GFP (Fig. 2f) than CD19 (Fig. 2g), which may be the result of CD19 CAR molecules binding to the trogocytosed CD19 in cis, blocking the anti-CD19 detection antibody and thereby leading to an underestimation of CD19 on the CAR T-cell surface. These data indicate that selective degradation of trogocytosed antigen is feasible in CAR T cells and that this approach can be used to explore the effects of CMT on CAR T-cell function.

It has previously been shown that FMC63 binding to CD19 in *cis*^37^ can cause persistent CAR signaling followed by T-cell exhaustion. We found that conventional FMC63 CAR T cells that had acquired CD19 during coculture with CD19-GFP-expressing K562 cells showed a twofold increase in the expression of the exhaustion markers LAG-3 and TIM-3 compared to negative control T cells expressing a CAR without a binding domain (∆scFv, Fig. 2h, i and Supplementary Fig. S2h, i). The expression of TAD_GFP_ prevented LAG-3/TIM-3 upregulation on these cells, demonstrating that increased exhaustion marker expression is the direct consequence of trogocytic antigen transfer to CAR T cells. CMT-induced fratricide and exhaustion could substantially limit long-term CAR T-cell antitumor activity. In a serial coculture stress test of CAR T-cell efficacy (Fig. 2j), we found that TAD_GFP_ substantially increased the ability of CAR T cells to control B-cell acute lymphoblastic leukemia (B-ALL, Fig. 2k).

These data demonstrate that CMT directly causes CAR T-cell dysfunction and fratricide, thereby limiting CAR T-cell persistence and antitumor activity.

### Real-time detection of CMT by luciferase complementation assay

**Fig. 3:**
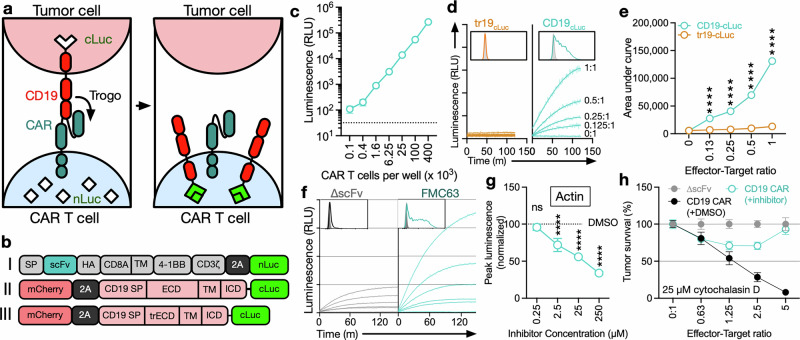
Luciferase complementation assay allows for real-time detection of trogocytosis. **a** Schema of the luciferase complementation assay (CompLuc). **b** Schema of constructs used for CAR T cells (I) and tumor cells (II/III) for the development of CompLuc. SP signal peptide, TM transmembrane region, trECD truncated extracellular domain, ECD extracellular domain, ICD intracellular domain. **c** Luminescence of nLuc + cLuc + CAR T cells. Data represent the mean ± S.D. of three technical replicates and are representative of two independent experiments. **d** CMT during coculture of FMC63 CAR T cells expressing nLuc with K562 cells expressing full-length (teal) or truncated (tr19, orange) CD19 fused to cLuc using CompLuc or flow cytometry after 1 h at the indicated effector-target ratios. CMT using CompLuc was measured for a total of 2.5 h at 1-min intervals. The data represent the best fit of three technical replicates. The boxed area represents a histogram of anti-CD19/FITC staining of CAR T cells at a 1:1 effector-target ratio after 1 h as measured by flow cytometry. **e** Area-under-curve quantification of luminescence data shown in Fig. 3D. Data represent the mean ± S.D. of three technical replicates. Statistical significance was determined by an unpaired *t*-test. **f** CMT during coculture of ∆scFv or FMC63 CAR T cells expressing nLuc with K562 cells expressing full-length CD19-cLuc using CompLuc or flow cytometry after 1 h at the indicated effector-target ratios. CMT using CompLuc was measured for a total of 2.5 h at 1-min intervals. The data represent the best fit of three technical replicates. The boxed area represents a histogram of anti-CD19/FITC staining of CAR T cells at a 1:1 effector-target ratio after 1 h as measured by flow cytometry. **d**–**f** Data are representative of at least three independent experiments. **g** Peak luminescence of FMC63 CAR T cells cocultured with K562 cells expressing CD19-cLuc at a 1:1 effector-target ratio as determined by CompLuc. CAR T cells were pretreated with cytochalasin D for 1 h at the indicated concentrations. Statistical significance was determined by one-way ANOVA. **h** Survival of Raji-Fluc cells after a 16-h coculture with FMC63 CAR T cells pretreated with the indicated concentrations of cytochalasin D. CMT values are normalized to a DMSO control. **g**, **h** Data represent the mean ± S.D. of three technical replicates. The data are representative of two independent experiments

Little is known regarding the molecular and cellular drivers of CMT, and to date, there are no high-throughput assays to systematically probe potential modulators of CMT as it occurs in real time. To address this, we developed a luciferase complementation assay for the real-time detection of CMT (CompLuc, Fig. 3a). In the CompLuc assay, luciferase complementation occurs following the transfer of CD19 fused to the C-terminal fragment of NanoLuc^38^ (cLuc) to CAR T cells, which express the complementary N-terminal NanoLuc (nLuc) fragment with high affinity for cLuc^38^ in the cytosol (Fig. 3b and Supplementary Fig. S3a, b). The CompLuc assay allows for sensitive detection of small numbers of nLuc^+^cLuc^+^ CAR T cells in cocultures (Fig. 3c). We demonstrate that CMT, as quantified by CompLuc, is antigen- (Fig. 3d, e) and CAR-dependent (Fig. 3f) and correlates with the effector-target ratio and antigen transfer as measured by flow cytometry (Fig. 3d–f).

To further validate the CompLuc assay, we determined the effect of an established modulator of CMT, the actin polymerization inhibitor cytochalasin D.^22,39^ Treatment of CAR T cells with cytochalasin D significantly reduced CMT in a dose-dependent manner, as measured by CompLuc (Fig. 3g), demonstrating the ability of the assay to identify modulators of CMT.

### Extracellular CTSB is a key driver of CMT

CMT may be the result of incomplete target cell killing followed by the endocytosis of target cell fragments, as suggested by the simultaneous effect of cytochalasin D on CMT (Fig. 3g) and CAR-mediated cytotoxicity (Fig. 3h). We hypothesized that while the molecular mechanisms driving target cell killing and trogocytosis may overlap, CMT could, in part, be mechanistically distinct from cytotoxic function.

**Fig. 4:**
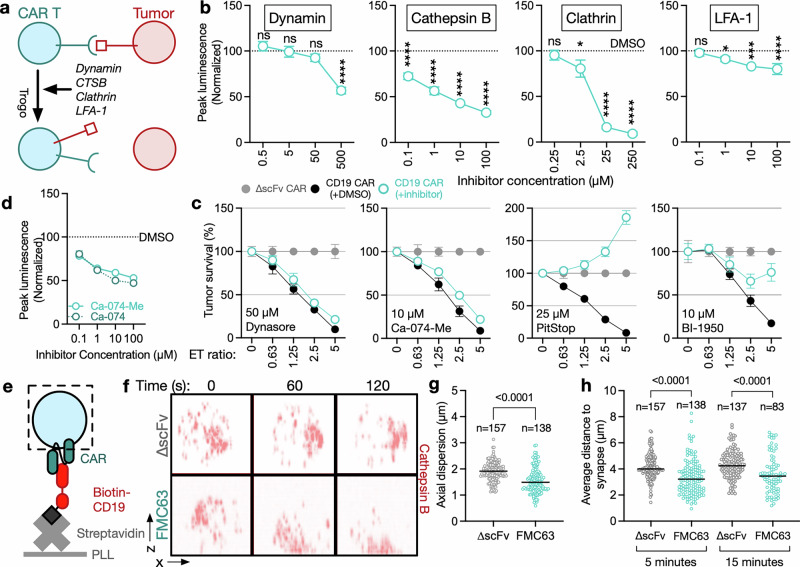
Extracellular CTSB is a driver of CMT. **a** Schema of potential modulators of CMT. **b** Peak luminescence as determined by CompLuc of FMC63 CAR T cells pretreated with inhibitors of the indicated proteins and then cocultured with K562 cells expressing CD19-cLuc at a 1:1 effector-target ratio. Values are normalized to DMSO/vehicle control. Data represent the mean ± S.D. of three technical replicates. Statistical significance was determined by one-way ANOVA. Data are representative of three independent experiments. **c** Survival of Raji-Fluc cells after 16 h of coculture with FMC63 CAR T cells treated with inhibitors of the indicated proteins at a single concentration. Tumor survival was measured using a luciferase-based cytotoxicity assay. Data represent the mean ± S.D. of three technical replicates. The data are representative of two independent experiments. **d** Peak luminescence as determined by CompLuc of FMC63 CAR T cells cocultured with K562 cells expressing CD19-cLuc at a 1:1 effector-target ratio. FMC63 CAR T cells were pretreated with Ca-074-Me (membrane permeable) or treated concurrently with Ca-074 (membrane impermeable) at the indicated concentrations. Values are normalized to DMSO/vehicle control. Data represent the mean ± S.D. of three technical replicates. Data are representative of three independent experiments. **e** Schematic of the imaging assay setup used to assess CTSB localization to the immune synapse. Biotinylated CD19 was immobilized on plate-bound NeutrAvidin. ∆scFv or FMC63 CAR T cells expressing CTSB-mCherry were added to slides and imaged using confocal microscopy. **f**–**h** ∆scFv or FMC63 CAR T cells expressing CTSB-mCherry were imaged by spinning disk confocal imaging after exposure to CD19 immobilized on glass slides at different time points. **f** Side (*x*–*z*) view of the distribution of CTSB-mCherry (red) at the indicated time points. The bottom of each box is aligned to the interface between the glass slide and the T cell. **g** Axial dispersion or **h** average distance of CTSB to the site of antigen contact. Data represent values from individual cells fixed 5 or 15 min after exposure to recombinant CD19. Numbers represent cells analyzed for each condition. **g**, **h** Statistical significance was determined by a two-tailed Student’s *t-*test. **f**–**h** Data are representative of at least two independent experiments

To identify functional systems that could be specifically involved in CMT but not cytotoxicity, we explored the effect of inhibiting various processes at the T-cell immune synapse on CMT^22,39–42^ (Fig. 4a). We found that inhibition of CTSB with the small molecule inhibitor Ca-074-Me in FMC63 CAR T cells reduced CMT in a dose-dependent manner, as measured by CompLuc (Fig. 4b), without substantially altering CAR T-cell cytotoxicity (Fig. 4c). When assessing the effect of CTSB inhibition on CMT in a solid tumor model of Ewing sarcoma, we found that CTSB inhibition in LINGO1-targeting CAR T cells also significantly reduced CMT (Supplementary Fig. S4). Using a membrane-impermeable inhibitor of CTSB (Ca-074), we found that inhibition of extracellular CTSB alone is sufficient to prevent CMT (Fig. 4d).

CTSB is a ubiquitously expressed cysteine protease that is primarily localized to lysosomal and endosomal compartments under physiological conditions, where it is primarily involved in protein degradation and turnover.^43,44^ However, CTSB is also found in the cytosol and exocytic vesicles, retains its catalytic activity at neutral pH,^45^, and has been shown to line exocytic granules of cytotoxic T lymphocytes.^46^ In addition to proteolytic degradation within the endosome and lysosome, CTSB has been implicated in the degradation and remodeling of components of the extracellular matrix, such as collagen and fibronectin, thereby promoting the invasion and metastasis of tumor cells.^45,47,48^ We hypothesize that CTSB may contribute similarly to the extraction of antigen-rich membrane fragments of target cells prior to their transfer to CAR T cells.

To further investigate the potential role of extracellular CTSB produced by CAR T cells in CMT, we next determined whether CTSB in CAR T cells is actively transported to the immune synapse upon antigen contact. To this end, we generated FMC63 CAR T cells expressing CTSB fused to mCherry and exposed these cells to recombinant CD19 (Fig. 4e). We assessed intracellular CTSB trafficking in these cells using live-cell confocal imaging after exposing CAR T cells to recombinant CD19 immobilized on a glass slide. We found that CTSB in FMC63 CAR T cells, but not in ∆scFv CAR T cells, rapidly localized to the site of antigen contact (Fig. 4f and Supplementary Fig. S1d), correlating with the timing of CMT as measured by CompLuc and flow cytometry (Fig. 3f). Specifically, following exposure to CD19, CTSB in FMC63 CAR T cells was significantly less dispersed throughout the cell (Fig. 4g), indicating coordinated CTSB trafficking and localization, and significantly closer to the site of antigen contact (Fig. 4h) compared to ∆scFv CAR T cells. These findings suggest that CTSB is actively and rapidly transported to the immune synapse upon antigen contact and that its subsequent secretion by CAR T cells contributes to CMT.

### Cystatin abundance controls CTSB activity and CMT

**Fig. 5:**
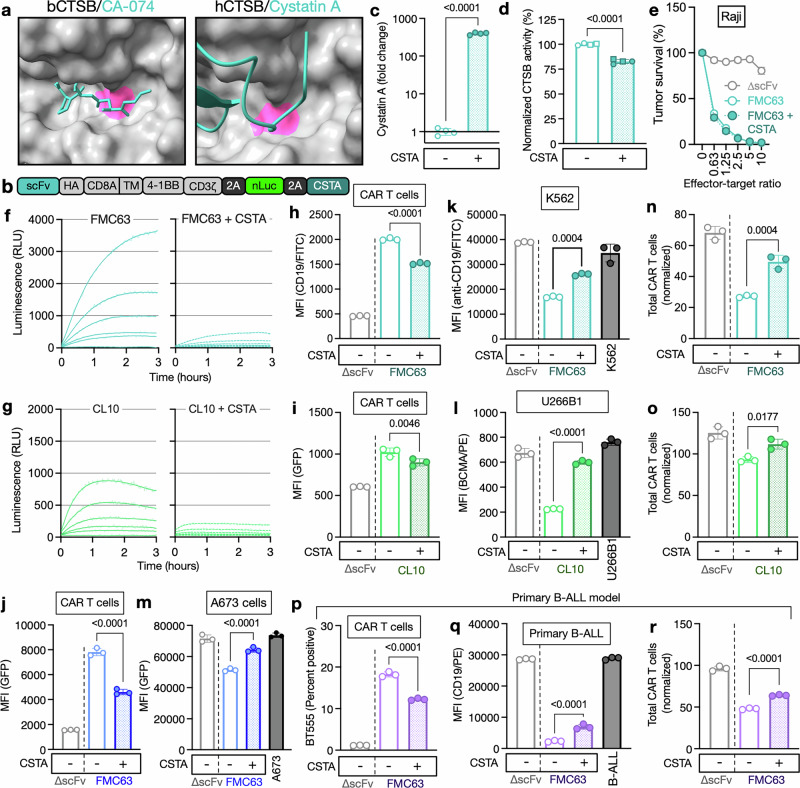
Cystatin abundance regulates CTSB activity and CMT. **a** Crystal structures of bovine cathepsin B (bCTSB) in complex with Ca-074 (left, PDB: 1QDQ) and human cathepsin B (hCTSB) in complex with cystatin A (right, PDB: 3K9M). The active site cysteine of CTSB is shown in pink; Ca-074 and cystatin A are shown in teal. **b** Schematic of the construct used for the production of FMC63-based CAR_CSTA_ T cells. **c** Amounts of CSTA in CAR or CAR_CSTA_ T-cell lysates, as measured by ELISA, are expressed as fold change of WT. Data represent mean ± S.D. of four technical replicates from two independent experiments. Statistical significance was determined by a two-tailed Student’s *t-*test. **d** CTSB activity in two different CD19-targeting (○ FMC63; □ CAT) CAR or CAR_CSTA_ T-cell products measured by fluorescence-based CTSB activity assay expressed as a percentage of WT. Data represent the mean ± S.D. of four replicates from two independent experiments. Statistical significance was determined by a two-tailed Student’s *t-*test. **e** Survival of Raji-Fluc cells after a 16-h coculture with FMC63 CAR or CAR_CSTA_ T cells at the indicated effector-target ratios. Data represent the mean ± S.D. of three technical replicates. Data are representative of at least three independent experiments using cells produced from three healthy donors. **f** CMT of FMC63 CAR or CAR_CSTA_ T cells as determined by CompLuc using K562 cells expressing CD19-cLuc after 3 h. Data are representative of three independent experiments using cells produced from three independent donors. **g** CMT in BCMA CAR or CAR_CSTA_ T cells as determined by CompLuc using K562 cells expressing BCMA-GFP-cLuc after 3 h. Data are representative of three independent experiments. **f**, **g** The data represent the best fit of three technical replicates. **h** CD19 transfer to CAR T cells following a 30-min coculture of FMC63 CAR or CAR_CSTA_ T cells with K562 cells expressing CD19-cLuc at a 0.5:1 effector-target ratio as determined by flow cytometry. Data are representative of at least three independent experiments using cells produced from three healthy donors. **i** BCMA-GFP transfer to CAR T cells following a 2-h coculture of BCMA CAR or CAR_CSTA_ T cells and K562 cells expressing BCMA-GFP-cLuc at a 1:1 effector-target ratio as determined by flow cytometry. Data are representative of three independent experiments. **j** GFP transfer to CAR T cells following a 1-h coculture with A673 cells engineered to express a CD19-GFP fusion protein at a 1:1 effector target ratio. **k** Amounts of CD19 on tumor cells following a 30-min coculture of FMC63 CAR or CAR_CSTA_ T cells and K562 cells expressing CD19-cLuc at a 0.5:1 effector-target ratio as determined by flow cytometry. Data are representative of at least three independent experiments using CAR T cells produced from three healthy donors. **l** Amounts of BCMA on U266B1 tumor cells following a 2-h coculture with BCMA CAR or CAR_CSTA_ T cells at a 1:1 effector-target ratio as determined by flow cytometry. Data are representative of three independent experiments. **m** Amount of GFP in A673 tumor cells following a 1-h coculture of FMC63 CAR or CAR_CSTA_ T cells and A673 cells expressing CD19-GFP at a 1:1 effector-target ratio as determined by flow cytometry. **j**, **m** Data are representative of two independent experiments using cells produced from three healthy donors. **n** Total CAR T cells following a 30-min coculture of FMC63 CAR or CAR_CSTA_ T cells and K562 cells expressing CD19-cLuc at a 0.5:1 effector-target ratio as determined by flow cytometry. Data are representative of at least three independent experiments using cells produced from three healthy donors. **o** Total CAR T cells following a 2-h coculture of BCMA CAR or CAR_CSTA_ T cells and K562 cells expressing BCMA-GFP-cLuc at a 1:1 effector-target ratio as determined by flow cytometry. Data are representative of three independent experiments. **n**, **o** CAR T-cell numbers are normalized to wells containing only CAR T cells using counting beads. **p** Membrane transfer to CAR T cells following a 1-h coculture of FMC63 CAR or CAR_CSTA_ T cells and primary B-cell acute lymphoblastic leukemia (B-ALL) cells at a 1:1 effector-target ratio as determined by flow cytometry. Circles indicate three technical replicates from a single patient B-ALL sample. **q** Amounts of CD19 on primary B-ALL cells following a 1-h coculture of FMC63 CAR or CAR_CSTA_ T cells and primary B-ALL cells at a 1:1 effector-target ratio as determined by flow cytometry. **r** Total CAR T cells following a 1-h coculture of FMC63 CAR or CAR_CSTA_ T cells and primary B-ALL cells at a 1:1 effector-target ratio as determined by flow cytometry. **q**, **r** Data are representative of three independent experiments using a single patient B-ALL sample. **h**–**r** Statistical significance was determined by one-way ANOVA

CTSB is ubiquitously expressed across tissues and cell types, and its activity is regulated through a network of endogenous protease inhibitors called cystatins.^49^ Cystatin A (CSTA) has been described as the main regulator of CTSB activity.^50^ Like Ca-074, CSTA inhibits CTSB through insertion of a hydrophobic wedge into the active-site cleft of CTSB^51^ (Fig. 5a). To determine whether CSTA abundance directly regulates CMT in CAR T cells, we engineered FMC63 CAR T cells to stably overexpress CSTA (CAR_CSTA_, Fig. 5b). We confirmed that CSTA overexpression led to significantly increased levels of CSTA protein in CAR T cells (Fig. 5c), which resulted in reduced CTSB activity (Fig. 5d) without altering in vitro CAR T-cell expansion (Supplementary Fig. S5a, b), phenotype (Supplementary Fig. S5c, d), or cytokine secretion (Supplementary Fig. S5e). It has previously been shown that CTSB^KO^ mice are viable, suggesting that loss of CTSB activity may not cause major toxicity^52–55^, but it is unknown whether overexpression of CSTA in T cells could cause toxicity. Because the homology between human and mouse CSTA is relatively limited (Supplementary Fig. S6a), we generated FMC63 CAR T cells overexpressing human CSTA (hCSTA) or mouse CSTA (mCSTA, Supplementary Fig. S6b, c). Mice treated with hCSTA or mCSTA CAR T cells (Supplementary Fig. S6d) did not show weight loss (Supplementary Fig. S6e) or changes in major tissue architecture (Supplementary Fig. S6f). When cocultured with tumor cells, CD19 CAR_CSTA_ cells showed equivalent short-term antitumor activity compared to conventional CAR T cells (Fig. 5e and Supplementary Fig. S7a), whereas BCMA CAR_CSTA_ cells showed slightly improved short-term antitumor activity (Supplementary Fig. S7b). When cocultured with CD19^+^ or BCMA^+^ K562 cells, we observed significantly reduced CMT in CAR_CSTA_ cells as measured by CompLuc (Fig. 5f, g and Supplementary Fig. S7c, d) despite comparable CAR T-cell activation (Supplementary Fig. S7e). CMT was similarly reduced when overexpressing a truncated CSTA_1–57_ containing the hydrophobic wedge (Supplementary Fig. S7f) or the related protein cystatin B^56^ (Supplementary Fig. S7g–i). CAR_CSTA_ T cells cocultured with relevant hematologic or solid tumor cell lines showed reduced antigen transfer to CAR T cells (Fig. 5h–j and Supplementary Fig. S7j–m) and reduced antigen loss on tumor cells (Fig. 5k-m, Supplementary Fig. S7n), suggesting inhibition of CMT at the antigen extraction step. This CSTA-mediated inhibition of CMT resulted in significantly increased CD19 and BCMA CAR T-cell numbers after short-term exposure to tumor cells in vitro (Fig. 5n, o and Supplementary Fig. S7o). To explore whether CSTA overexpression would be effective in a primary tumor cell model, we cocultured CAR or CAR_CSTA_ T cells with primary tumor cells from a patient with B-ALL and found that, in this setting, CSTA overexpression significantly reduced CMT (Fig. 5p, q), improved tumor control (Supplementary Fig. S7p), and increased CAR T-cell numbers (Fig. 5r). These data demonstrate that in short-term cocultures with tumor cells, CSTA overexpression significantly reduces CMT and increases CAR T-cell survival.

**Fig. 6:**
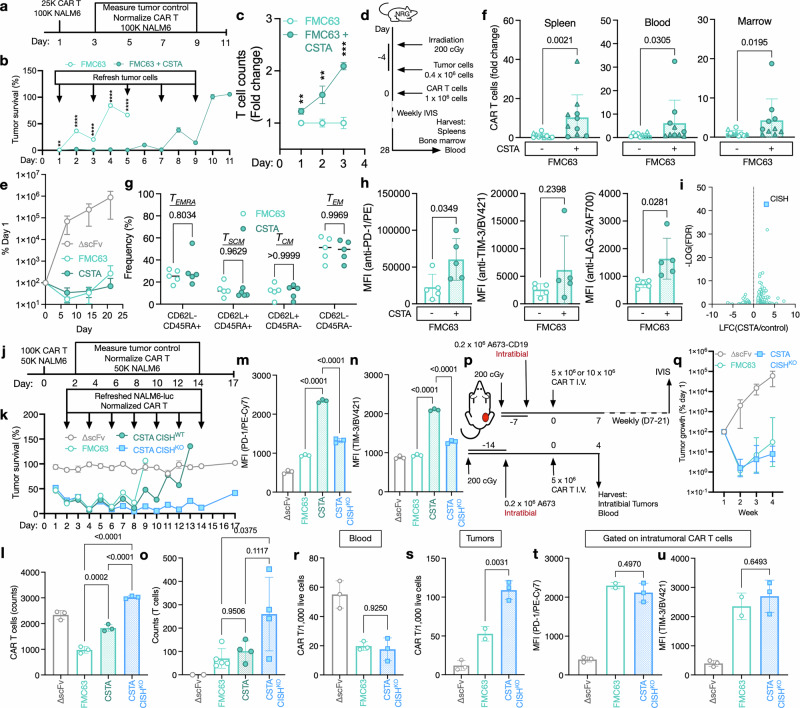
Cystatin overexpression enhances long-term CAR T-cell persistence. **a** Schema of the in vitro serial cytotoxicity experiment to determine long-term CAR T-cell persistence and antitumor activity. **b** Survival of CD19-GFP-expressing NALM6-Fluc cells at a 0.5:1 effector-target ratio using a luminescence-based cytotoxicity assay. Data indicate the mean ± S.D. from three technical replicates. Tumor survival was normalized to untreated tumor cells. The data are representative of two independent experiments. Statistical significance comparing FMC63 CAR and CAR_CSTA_ T cells was determined by multiple two-tailed Student’s *t* tests**. c** FMC63 CAR and CAR_CSTA_ T-cell counts on days 1–3 of the in vitro serial coculture described in Fig. 6A. Data represent the mean ± S.D. of 3 technical replicates and are representative of two independent experiments. Statistical significance comparing FMC63 CAR and CAR_CSTA_ T cells was determined by multiple two-tailed Student’s *t* tests. **d** Schema of the in vivo experiment to measure NALM6 tumor control and CAR T-cell persistence. **e** Tumor burden in mice bearing systemic NALM6-Fluc tumors and treated with ∆scFv or FMC63 CAR or CAR_CSTA_ T cells as determined by an in vivo imaging system (IVIS). Data represent the mean ± S.D. of 5 mice per group. **f** Quantification of CAR T cells in murine spleen, peripheral blood, and bone marrow as determined by flow cytometry. Data represent the mean ± S.D. of 10 mice pooled from two independent experiments, with experiments denoted by symbols. Data were normalized to the average of the respective experiment’s CSTA^–^ condition and are expressed as fold change. Statistical significance was determined by a two-tailed Student’s *t-*test. **g** Phenotype of FMC63 CAR/CAR_CSTA_ T cells from murine blood as determined by flow cytometry. Statistical significance was determined by two-way ANOVA. **h** PD-1, TIM-3, and LAG-3 expression on FMC63 CAR or CAR_CSTA_ T cells isolated from murine blood as determined by flow cytometry. Statistical significance was determined by a two-tailed Student’s *t-*test. **g**, **h** Data represent the mean ± S.D. of 5 mice per group. **i** Differential mRNA expression in sorted FMC63 CAR and CAR_CSTA_ T cells from two independent productions at the end of manufacturing as determined by bulk RNA sequencing. **j** Schema of the in vitro serial cytotoxicity experiment to determine long-term CAR T-cell persistence and antitumor activity. **k** Survival of NALM6-Fluc cells at a 2:1 effector target ratio using a luminescence-based serial cytotoxicity assay. On Days 2, 4, 6, 8, 10, 12, and 14, CAR T cells were normalized, and fresh tumor cells were added. **l** Total CAR T cells on day 10 of the in vitro serial coculture described in Fig. 6J. **m** PD-1 expression on CAR T cells on day 5 of in vitro serial coculture, as described in Fig. 6J. **n** TIM-3 expression on CAR T cells on day 5 of in vitro serial coculture, as described in Fig. 6J. **k**–**n** Data represent the mean ± S.D. of three technical replicates and are representative of two independent experiments. **o** Quantification of CAR T cells in bone marrow 28 days after intravenous treatment with the indicated CAR T-cell conditions as determined by flow cytometry. Data represent the mean ± S.D. of 2–5 mice per group. Statistical significance was determined by one-way ANOVA. **p** Schema of the in vivo experiment to measure solid tumor control and CAR T-cell infiltration into solid tumors. **q** Tumor burden in mice bearing intratibial CD19-expressing A673-Fluc tumors and treated intravenously with ∆scFv or FMC63 CAR CISH^WT^ or CAR_CSTA_ CISH^KO^ T cells as determined by an IVIS. Data represent the mean ± S.D. of 3–4 mice per group. **r** Quantification of CAR T cells per 1000 acquired live cells in peripheral blood four days after treatment with the indicated CAR T cell conditions as determined by flow cytometry (*n* = 2–3 per group). Statistical significance was determined by one-way ANOVA. **s** Quantification of CAR T cells per 1000 acquired live cells in intratibial A673 tumors four days after treatment with the indicated CAR T-cell conditions as determined by flow cytometry (*n* = 2–3 per group). Statistical significance was determined by one-way ANOVA. **t** PD-1 and **u** TIM-3 levels on intratumoral CAR T cells four days after CAR T-cell injection as determined by flow cytometry. Statistical significance was determined by one-way ANOVA. **r**–**u** Data represent the mean ± S.D. of 2–3 mice per group

We hypothesized that this effect on CAR T-cell survival in short-term cocultures could also lead to increased long-term CAR T-cell persistence and antitumor activity of CAR_CSTA_ T cells. We therefore next performed a serial cytotoxicity assay to determine CAR_CSTA_ T-cell antitumor activity over an extended period of time (Fig. 6a). We found that CAR_CSTA_ T cells significantly prolonged tumor control (Fig. 6b) and observed increased CAR T-cell numbers over time (Fig. 6c). In an in vivo B-ALL tumor model (Fig. 6d), we observed tumor control in all groups treated with CD19-targeting CAR T cells (Fig. 6e and Supplementary Fig. S8a) and again found significantly increased CAR_CSTA_ T-cell numbers across tissues (Fig. 6f). When analyzing the phenotype of these persisting CAR T cells, we found that CSTA overexpression did not significantly alter the T-cell subset composition (Fig. 6g). We next explored the expression of exhaustion markers in CAR T cells from in vitro serial cytotoxicity and in vivo experiments. We found that despite their substantially increased persistence in both experiments, CAR_CSTA_ cells showed significantly increased levels of exhaustion (Fig. 6h and Supplementary Fig. S8b). To explore potential causes of this increased exhaustion, we next performed bulk RNA sequencing of FMC63 CAR and CAR_CSTA_ T cells at the end of production. We found that CSTA overexpression resulted in the spontaneous upregulation of cytokine-inducible SH2-containing protein (CISH), which has previously been implicated as a key driver of CAR T-cell exhaustion (Fig. 6i). To determine whether this upregulation of CISH could have caused the increased exhaustion of CAR_CSTA_ T cells, we next established an efficient CRISPR/Cas9-mediated CISH knockout (Supplementary Fig. S8c, d). CISH^KO^ did not result in changes in T-cell subset composition at the end of production (Supplementary Fig. S8e) and did not affect the inhibition of CMT by CSTA overexpression (Supplementary Fig. S8f–h), indicating that the effect of CSTA on CMT is not mediated by CISH. We next performed a serial cytotoxicity assay using CAR_CSTA_ cells with and without CISH^KO^ (Fig. 6j) and found that CISH^KO^ significantly prolonged tumor control and further increased CAR T-cell persistence over time (Fig. 6k, l). In addition, CISH^KO^ almost entirely prevented exhaustion in CSTA-expressing CAR T cells in vitro (Fig. 6m, n), indicating that CSTA-induced upregulation of CISH caused increased levels of exhaustion in CAR_CSTA_ cells. We validated this finding in the same in vivo model described above and again observed complete tumor control and increased persistence in CAR_CSTA_ CISH^KO^ CAR T cells (Fig. 6o and Supplementary Fig. S8i). We next hypothesized that CMT could be more pronounced in the solid tumor setting due to the higher density of tumor cells, leading to more extensive antigen transfer and fratricide. We therefore established a solid tumor in vivo model by intratibial implantation of A673 Ewing sarcoma cells engineered to express CD19 (Fig. 6p). This model was characterized by rapid localized tumor growth and comparable tumor control by systemically injected wild-type FMC63 CAR T cells as well as CAR_CSTA_ CISH^KO^ CAR T cells (Fig. 6q and Supplementary Fig. S8j). In this model, we found that CSTA expression did not alter CAR T-cell numbers in the peripheral blood (Fig. 6r) but significantly increased intratumoral CAR_CSTA_ T-cell numbers, indicating that CSTA overexpression does not negatively affect tumor infiltration by CAR T cells (Fig. 6s) and instead efficiently prevents intratumoral CAR T-cell fratricide. The exhaustion and phenotype of CAR_CSTA_ CISH^KO^ cells were comparable to those of FMC63 CAR T cells, indicating that CISH^KO^ robustly prevents CSTA-mediated exhaustion in solid and hematologic settings (Fig. 6t, u and Supplementary Fig. S8k).

Taken together, these data indicate that CSTA overexpression leads to significantly reduced CMT, as well as increased long-term CAR T-cell persistence and in vitro tumor control. CSTA-mediated induction of exhaustion via CISH could be overcome by simultaneous CISH^KO,^ further enhancing functional CAR T-cell persistence.

## Discussion

CAR T cells have revolutionized cancer immunotherapy, but most patients receiving CAR T-cell therapy still relapse within 5 years of treatment.^12,57,58^ Several mechanisms driving relapse after CAR T-cell therapy have been described, including CAR T-cell-mediated trogocytosis (CMT). CMT, the extraction of tumor antigen from the tumor cell and its subsequent incorporation into the CAR T-cell membrane, is modulated by CAR affinity for target antigen,^23^ actin rearrangement,^22,39^ cholesterol metabolism,^59^ and the CAR transmembrane domain.^60^ CMT has previously been shown to result in the emergence of antigen-negative tumor cells^22–24^ and has been hypothesized to cause CAR T-cell fratricide as well as CAR T-cell exhaustion through persistent cis signaling.^37^ Previous studies have demonstrated the presence of tumor antigen on CAR T cells following coculture with tumor cells, and that the presence of trogocytosed antigen correlates with increased apoptotic markers and exhaustion.^22–24^ However, it remained unknown whether this T-cell dysfunction was indeed caused by CMT. In this study, we sought to determine whether the presence of trogocytosed antigen causes CAR T-cell dysfunction and to uncover molecular modulators of CMT to improve our understanding of the trogocytic process.

In this study, we demonstrate that CAR T-cell fratricide and exhaustion can be reversed through selective degradation of trogocytosed antigen on CAR T cells, indicating that trogocytosed antigen is indeed directly causing CAR T-cell dysfunction. We show that this TAD approach is feasible when targeting an antigen-GFP fusion protein as well as the unmodified endogenous antigen (CD19) by using endogenous protein domains binding to the target antigen’s intracellular domain. The TAD protein targeting endogenous CD19 (TAD_CD19_) relies on the established high-affinity interaction of the Lyn kinase SH2 domain with the intracellular domain of CD19.^36^ However, SH2 domains can be involved in multiple signaling pathways, with their activity being highly context dependent.^61–63^ It is therefore possible that TAD_CD19_ also binds to proteins other than CD19. Because of this possibility, we mainly relied on the use of TAD_GFP_, which relies on a validated, highly specific protein‒protein interaction and is unlikely to result in off-target effects.^32^ If TAD-mediated targeting of an endogenous antigen is desired in the absence of target-specific endogenous binding domains, protein binders could be generated de novo, analogous to the GFP-specific VHH domain used in this study. Future work focusing on TAD-based approaches using endogenous binding domains will have to carefully consider potential off-target reactivity.

In this study, we found that the proteolytic activity of the cysteine protease CTSB produced by CAR T cells is essential for CMT but dispensable for tumor cell killing. We demonstrate that CTSB produced by CAR T cells rapidly localizes to the immune synapse upon antigen stimulation and that extracellular CTSB confers antigen extraction from tumor cells. The role of extracellular CTSB in the processing of cellular material is in line with prior studies describing the involvement of tumor-derived CTSB in the degradation and remodeling of components of the extracellular matrix, enabling tumor metastasis.^45,47,48^ We further show that CTSB activity in CAR T cells is tightly regulated through the abundance of cystatins, a class of endogenous protease inhibitors.^49,51^ We found that overexpression of both cystatin A and cystatin B potently inhibited CTSB in CAR T cells. While the small molecule inhibitors used throughout this study have been shown to be specific for CTSB,^64,65^ the more robust inhibition of CMT by cystatins, which are able to inhibit a range of cathepsins, including cathepsin L and H, could indicate a role of other cathepsins in CMT.^49,51^

In principle, constitutive overexpression of CSTA to limit CMT could cause toxicity through systemic inhibition of CTSB or other proteases.^66^ However, it has been shown previously that CTSB^KO^ mice are viable and appear indistinguishable from CTSB^WT^ mice.^52–55^ In addition, we found that mice treated with CAR_CSTA_ T cells exhibit no overt signs of toxicity. While constitutive overexpression of CSTA appears to be relatively safe, additional work will need to be performed in relevant model systems to confirm our initial observations. If toxicity is observed, the safety of CSTA overexpression could be increased by using a conditional expression system that initiates CSTA expression upon CAR target recognition or recognition of a separate tissue-specific antigen.^67,68^

In this study, we specifically focused on the role of CAR T-cell-derived factors in CMT. However, CTSB is expressed by both T cells and tumor cells. Using the covalent small molecule CTSB inhibitor Ca-074-Me, we show that specific inhibition of CAR T-cell-derived CTSB directly causes CMT. However, it is possible that tumor-derived CTSB also contributes to this process, and further work is needed to comprehensively explore this possibility.

Increased levels of cystatins other than CSTA, such as cystatin F, a type II cystatin and inhibitor of cathepsins C, L, and H, have been found to decrease cytotoxicity in NK cells and T cells,^69,70^ raising the question of how CSTA overexpression may affect CAR T-cell function and tumor control. In this study, we observed no differences in short-term in vitro cytotoxicity and cytokine secretion between FMC63 CAR and CAR_CSTA_ T cells. However, we observed substantially increased long-term tumor control by CAR_CSTA_ T cells in vitro, which was also associated with significantly increased CAR T-cell numbers in hematologic and solid tumor models in vitro and in vivo. Increased tumor control by CAR_CSTA_ T cells is in line with previous studies, which showed that inhibiting CMT using inhibitory CARs,^24^ combinatorial targeting,^22^ or altering cholesterol metabolism^59^ can increase antitumor activity. However, in long-term in vitro and in vivo experiments, we observed significant upregulation of exhaustion markers on CAR_CSTA_ T cells. Analyzing the preinfusion product, we found that CAR_CSTA_ T cells showed spontaneous upregulation of CISH, a known negative regulator of T-cell activation.^71,72^ By including a CISH knockout step during CAR_CSTA_ production, we were able to show that CSTA-induced exhaustion was mediated by CISH, which is in line with recent studies showing that CISH can be a major driver of CAR T-cell exhaustion in some contexts.^72,73^ In contrast to our long-term in vitro cytotoxicity data, we did not observe significantly increased in vivo antitumor activity by CSTA-overexpressing CAR T cells compared to wild-type CAR T cells, even following CISH knockout. The group sizes used in this study may have been too small to detect a significant difference between these groups. It is also possible that more physiologically relevant or immunocompetent mouse models will be needed to validate the findings observed in vitro with the same CAR T-cell products.

Our work serves as an initial proof-of-concept showing that protease inhibition is an effective approach to limit CMT, but additional work will be needed to better understand how CSTA alters CISH expression and whether it is possible to further separate the beneficial effects of CSTA overexpression from those conferring increased exhaustion.

In addition to investigating the effects of CMT on CAR T-cell persistence and function, we show here that antigen density is substantially lowered on tumor cells through CMT and that CMT is detectable in patients treated with CAR T cells. However, it remains unclear how much transient antigen loss observed after CMT contributes to clinical antigen escape. Monitoring CMT with high temporal resolution in vivo through continuous sampling could help to answer this question by elucidating the extent of CMT-mediated antigen loss. In addition, high-resolution imaging of in vitro cocultures with tumor cells and CAR T cells may allow visualization of tumor cells following CMT to determine whether antigen loss protects these cells temporarily from CAR T-cell-mediated killing.

Trogocytosis is a highly variable phenomenon and can be affected by several factors, including tumor antigen density,^22^ CAR affinity,^23^ CAR structure,^22,60^ and target cell type. We observed substantial variability in CMT when analyzing the peripheral blood of patients receiving CAR T-cell therapy, and recent work also shows variability in CMT in mice.^60^ It remains an open question why CMT varies between patients, and larger clinical immunomonitoring trials will be needed to conclusively address this question.

Our study primarily focused on approaches targeting the two clinically validated CAR T-cell antigens CD19 and BCMA approved for the treatment of several hematologic malignancies; however, we also show that CMT can be observed in solid malignancies when targeting different, previously validated CAR antigens. While CMT appears to occur across CAR target antigens, it remains an open question whether transfer of functionally essential and/or tumorigenic proteins, the loss of which would result in rapid target cell death, would be equally susceptible to this process. In the solid tumor setting, exhaustion-driven T-cell dysfunction is generally more pronounced^,^^74,75^ and CMT may play a key role due to a denser tumor microenvironment and limited T-cell mobility, which may favor more extensive intratumoral fratricide. High-resolution intratumoral imaging may provide critical insights into CMT dynamics to inform future strategies to reduce the impact of this process.

In conclusion, we show that CMT directly causes CAR-mediated fratricide and exhaustion and that CTSB is essential for CMT. CTSB activity is controlled by the abundance of endogenous human cystatins, and cystatin overexpression results in significantly reduced CMT, fratricide, and exhaustion and increased CAR T-cell persistence and antitumor activity. CSTA overexpression is an effective approach to limit the negative consequences of CMT but requires additional engineering, such as CISH knockout, to prevent CSTA-mediated CAR T-cell exhaustion. Our findings provide evidence that CMT is mechanistically distinct from cytotoxic function in CAR T cells and support the rational targeting of this resistance mechanism in CAR T cells.

## Materials and methods

### Study approval

All recombinant DNA and biosafety work was approved by the institutional biosafety committees at the University of Maryland, Baltimore (protocol IBC-6040). Animal experiments were approved by the Institutional Animal Care and Use Committee at the University of Utah (protocol 18-1104). Patient samples were collected under the Institutional Review Board (IRB)-approved protocol 2043GCCC (IRB H0091736, PI D. Atanackovic) after obtaining informed consent.

### Cell lines and primary human cells

Raji, NALM6, Daudi, DB, Toledo, MM.1S, RPMI8226, U266B1, K562, A673, TC-71, and Phoenix-AMPHO cells were purchased from the American Type Culture Collection (ATCC) and cultured according to ATCC instructions. Lenti-X 293T cells were purchased from Takara and cultured according to the manufacturer’s instructions. Cell lines were authenticated by their respective suppliers. Healthy donor buffy coats were obtained from the New York Blood Center. PBMCs from healthy donors were isolated from buffy coats by density gradient using FillPaque (GE) as previously described in refs. ^27,30^ Primary human T cells were cultured in AIM-V medium (Invitrogen 12055-083) supplemented with 5% human serum (Sigma H4522-100ML), 1% Pen/Strep (Thermo Fisher 15140-122) (T-cell media), and 40 IU IL-2 (R&D Systems #202-IL-10). All cells were cultured at 37 °C and 5% CO_2_.

### Vector constructs

All vectors generated for this study were produced by Twist Biosciences. All CAR constructs contain the CD8α hinge/transmembrane domain, 4-1BB costimulatory domain, and CD3z domain. CAR constructs were generated using existing binders for CD19 (clone: FMC63^76^ or CAT^77^), FolRα (clone: C4),^78^ BCMA (Carvykti and Abecma),^79^ GD2^80^, and LINGO1 (clone: Li82).^31^ For some constructs, the full-length sequence of human cystatin A (UniProt, P01040) was synthesized and cloned downstream of the respective CAR and nLuc fragment separated by a P2A sequence (Twist Bioscience). DNA was isolated using an Endofree Plasmid Maxi Kit (Qiagen 12362) following the manufacturer’s protocol. Plasmid concentration was measured using a NanoDrop One instrument (Thermo). All DNA constructs were stored at −20 °C.

### Patient samples

Whole blood was drawn from patients receiving CAR T-cell therapy 7–28 days after CAR T-cell injection. Limited numbers of patient samples were available for this project due to competition for the same types of samples by other studies at our institution, including multiple large-scale CAR T-cell immunomonitoring studies. PBMCs were isolated by density gradient using Ficoll Paque as previously described.^27,30^ PBMCs were stained with anti-hCD3, anti-hCD4, anti-hCD8, anti-CAR (Miltenyi #130-127-342), anti-hCD19, anti-hCD27, anti-hCD137, and 7-AAD and analyzed by flow cytometry.

### Gamma retrovirus production

Gamma retrovirus was produced using Phoenix-AMPHO cells (ATCC, catalog no. CRL-3213). Phoenix-AMPHO cells were transiently transfected with 16 µg of plasmid DNA using Opti-MEM Reduced Serum Medium (Thermo Fisher, catalog no. 31985070) and Lipofectamine 2000 (Invitrogen 11668-019) according to the manufacturer’s instructions. During transfection, cells were cultured in Dulbecco’s modified Eagle’s medium (DMEM, Thermo Fisher 11995073) supplemented with 10% FBS. Virus-containing supernatant was filtered using Steriflip™ Sterile Disposable Vacuum Filter Units (Millipore Sigma SEIM003M00). The virus was concentrated using a Retro-X Concentrator (Takara, 631456). The following day, the concentrated virus was centrifuged at 1500×*g* for 45 min at 4 °C. The supernatant was removed, and the concentrated virus was resuspended in 1.5 mL complete T-cell media.

### Transgenic T-cell production and expansion

CAR T cells were generated as previously described in refs. ^23,27,30^ Buffy coats from three healthy donors were obtained from the New York Blood Center, and PBMCs were isolated using Ficoll-Paque and cryopreserved until use. PBMCs were thawed and cultured overnight in complete T-cell media. PBMCs were stimulated for 2 days with anti-CD3/anti-CD28 T-cell activation beads (Thermo, catalog no. 11131D) in the presence of interleukin-2 (IL-2; 40 IU/mL; R&D Systems, catalog no. 202-IL-010) in complete T-cell media and incubated at 37 °C and 5% CO_2_. Bead-stimulated cells were transferred to RetroNectin-coated (Takara) virus–containing plates and incubated overnight. Transduction was repeated the next day before counting and diluting cells to 0.4 × 10^6^ cells/mL. After the second transduction, cells were grown for an additional 7 days before removing beads using a DynaMag-15 magnet (Thermo Fisher Scientific). IL-2 was replenished every 2 days to 40 IU/mL. Cells were frozen in 90% fetal bovine serum (FBS)/10% dimethyl sulfoxide and stored in liquid nitrogen. CAR T-cell transduction efficiency and phenotype were determined by flow cytometry. CAR T cells were washed with FACS buffer and stained with antibodies targeting hCD3, HA, hCD4, hCD8, hCD95, hCD62L, and hCD45RA. Because BCMA CAR_CSTA_ T cells showed substantially lower transduction efficiency than conventional BCMA CAR T cells, after production, we FACS sorted both products following anti-HA (CAR) staining using a FACSAria cell sorter (BD) prior to subsequent functional assays.

To generate CISH^KO^ CAR T cells, on day 5, anti-CD3/CD28 T activator beads (Thermo Fisher) were removed from the transduced cells by magnetic separation. CRISPR ribonucleoprotein (RNP) particles containing 25 μg Cas9 only (mock/WT) or Cas9 and 150 pmol synthetic TrueGuide gRNA (CISH target sequence: 5’-GACAGCGUGAACAGGUAGCU-3’) were prepared according to the manufacturer’s instructions (Thermo). A total of 3 × 10^6^ cells were electroporated in the presence of CRISPR-RNPs using the NEON electroporation system (Thermo) using the following settings: 1600 V, 10 ms, 3 pulses. Following electroporation, the cells were transferred to prewarmed media and incubated for 2 h at 37 °C. Following incubation, new anti-CD3/CD28 T-cell activation beads were added to the electroporated cells at a ratio of 3:1, and the cells were grown for an additional 6 days before removing the beads using a DynaMag-15 magnet (Thermo Fisher). IL-2 was replenished every 2 days to 40 IU/mL. Cells were frozen in 90% FCS/10% DMSO and stored in liquid nitrogen.

### Imaging substrate preparation

Eight-well chambers (Cellvis, catalog no. C8-1.5H-N) were used for all experiments. For CAR T-cell activation on CD19-coated surfaces, 8-well chambers were coated with 0.01% poly-L-lysine (PLL) solution diluted in distilled water for 10 min at room temperature. PLL was aspirated from each well, and the chambers were allowed to air-dry for 1 h at 37 °C. PLL-coated dishes were then incubated overnight at 4 °C with a 10 μg/mL solution of NeutrAvidin (Thermo Scientific, catalog no. 31000) in 1× Dulbecco’s phosphate-buffered saline (DPBS). After overnight incubation, coated wells were washed with 1× DPBS at room temperature and incubated at 37 °C with a 10 μg/mL solution of biotinylated human CD19 protein (ACRO Biosystems catalog no. CD9-H82E9) in DPBS for 2 h at 37 °C. Prior to the experiment, coated wells were washed three times with RPMI 1640 phenol red-free imaging medium. For coculture experiments, 8-well chamber wells were incubated with 10 μg/mL fibronectin (Millipore Sigma, catalog no. 34-163-11MG) in DPBS at room temperature for 1 h prior to seeding 293T cells stably expressing CD19-GFP. 293T cells were seeded at a concentration of 5 × 10^5^ cells per well in complete growth media, followed by an overnight incubation at 37 °C and 5% CO_2_. Prior to imaging, the wells were washed three times with warm complete imaging media consisting of a 1:1 ratio of RPMI 1640 supplemented with 5% FBS and DMEM supplemented with 10% FBS. The washing process was performed thrice to ensure the removal of any residual media while leaving a known volume in the wells.

### Confocal microscopy

Confocal microscopy was conducted using an inverted microscope (Nikon Ti-E PFS, Nikon Inc.) equipped with a 100× silicone objective lens. Imaging was performed with a Prime BSI camera (Photometrics). Image acquisition protocols were managed using Nikon Elements software, and images were cropped in FIJI for further analysis. All live cell imaging was performed with imaging chambers placed in a stage-top Okolab Incubator (Okolab S. R. L.) preequilibrated to 37 °C with 5% CO_2_. For live cell imaging on glass, activated CAR T cells suspended in RPMI 1640 medium supplemented with 5% FBS were deposited onto CD19-coated surfaces. Imaging was started between 3 and 6 min after CAR T cells expressing CTSB-mCherry were added to a biotin-CD19-coated coverslip. For each well, time-lapse images of a 3D volume (planes with a z spacing of 0.3 μm to span the cell from the top to the bottom) were acquired every 3 min for 60 min. For coculture experiments, imaging was started between 3 and 6 min after CAR T cells expressing CTSB-mCherry were dropped onto a layer of 293 T cells expressing CD19-GFP seeded on a coverslip at a concentration of 7 × 10^4^ cells per drop. Brightfield imaging was used to identify cells attached to the apical surface of HEK293 cells. The synaptic plane between a CAR-T-cell and a HEK293 cell was identified and designated the home plane for the acquisition of Z-stack time-lapse movies. Two-channel images using 488 nm and 561 nm lasers (for GFP and mCherry imaging, respectively) were acquired every 3 min for 1 h and 15 min utilizing a *Z*-spacing of 0.3 or 0.6 μm, with brightfield images taken at the home plane.

Image analysis was carried out in MATLAB (MathWorks, Inc.) using custom scripts. The plane of the synapse was determined using the actin channel. After background subtraction, axial intensity gradients are estimated for every voxel of sufficient intensity within the ROI. Below the cell, these gradients are typically positive due to the Airy pattern of the PSF. The synapse is taken as the first plane for which the gradients are no longer consistently positive.

### Estimating the average distance of CTSB to the synapse

The center of fluorescence (COF) of CTSB is defined similarly to the center of mass. The voxel positions are weighted by CTSB intensity after background subtraction and thresholding to obtain the COF. The average CTSB distance is then calculated from the axial (*z*) distance of the CTSB COF to the plane of the synapse.

### Characterizing CTSB axial dispersion

The axial dispersion is an estimate of the average distance of CTSB molecules to the COF. The axial (*z*) distance of each voxel to the COF is determined, and the axial dispersion is defined as the average of these distances weighted by CTSB intensity after background subtraction and thresholding. Voxels with sufficiently low signals do not contribute to the calculation due to the thresholding procedure.

### CTSB clustering at the synapse

The pair autocorrelation function g(r) of CTSB is computed at the synapse using the actin channel as a mask.^81^ The actin channel is segmented by smoothing with a Gaussian filter, generating an initial mask by applying k-means clustering (*k* = 2) after a log transformation, and then applying morphological operations to connect and smooth the initial mask. The clustering coefficient, *g*_ave_, is computed by averaging over all radial bins with 0.25 m or 0.5 m.

### CompLuc-based trogocytosis assay

Transduced, cryopreserved nLuc+ CAR T cells were thawed and cultured in complete T-cell media supplemented with 40 IU IL-2 for 48 h prior to use. nLuc-expressing CAR T cells were cocultured with CD19-cLuc-expressing K562 tumor cells at the specified effector-target ratios. CAR T cells and tumor cells were resuspended in Opti-MEM reduced serum media. Live Cell Substrate (Promega, catalog no. N2011) was prepared according to the manufacturer’s instructions. K562 tumor cells and prepared live cell substrate were added to wells of a black 96-well plate, and luminescence was measured to assess baseline luminescence. CAR T cells were added to the appropriate wells, and luminescence was measured every minute for 3 h at 37 °C. Luminescence was measured using a Spark multimode plate reader (Tecan).

### Flow cytometry-based trogocytosis assay

A flow cytometry-based trogocytosis assay was used to confirm the results observed in CompLuc, to assess CD19 or BCMA levels on CAR T cells and tumor cells, and to quantify CAR T cells. Target cells (5 × 10^4^) were seeded in the wells of a 96-well round-bottom plate. Various ratios of CAR T cells produced from one of three healthy donors were cocultured with target cells for 1 h at 37 °C and 5% CO_2_. Following coculture, cells were resuspended by gentle pipetting and transferred to wells of a 96-well V-bottom plate for washing and staining. Cells were stained with Zombie violet or Zombie NIR fixable viability dye and antibodies against hCD3, HA, hCD19, or BCMA (Supplementary Table S1). When assessing exhaustion, cells were stained with antibodies targeting PD-1, LAG-3, and TIM-3. When assessing phenotype, cells were stained with antibodies targeting CD45RA and CD62L. Accucheck counting beads (Life Technologies) were added to the cells for normalization. Samples were acquired on an LSR II flow cytometer (BD) or an Aurora full-spectrum flow cytometer (Cytek).

### Luciferase-based cytotoxicity assay

To determine in vitro CAR T-cell cytotoxicity, cell lines (Raji, NALM6, Daudi, Toledo MM.1S, RPMI8226, U266B1) were transduced with pHIV-Luc-ZsGreen lentivirus and sorted on a FACS Aria flow cytometer (BD) for GFP expression. Target cells (3 × 10^4^) were seeded in the wells of a 96-well round-bottom plate. CAR T cells from one of three healthy donors were cocultured with target cells at the indicated effector-target ratios and incubated for 16 h at 37 °C and 5% CO_2_. Following incubation, 80 μL of supernatant was harvested from each well. Cells were suspended by gentle pipetting, and 100 μL was transferred to a 96-well black flat-bottom plate. D-Luciferin (Gold Biotechnology, catalog no. LUCNA-2G) at 150 μg/mL was added to the cells and incubated for 5 min at 37 °C. Luminescence was determined on a Spark multimode plate reader (Tecan).

### Serial coculture repeat stimulation assay

To determine the long-term in vitro control and exhaustion of CAR T cells, luciferase-expressing tumor cells were plated at 5 × 10^4^ cells/well. CAR T cells were cocultured at a defined effector-target ratio and incubated for 24–48 h at 37 °C and 5% CO_2_. Following incubation, the cells were transferred to a 96-well black flat-bottom plate. D-luciferin was added to cells, and luminescence was determined on a Spark multimode plate reader (Tecan). After measuring luminescence, the cells were washed with FACS buffer and stained with Zombie NIR fixable viability dye (Biolegend) and antibodies targeting CD19, HA, PD-1, TIM-3, CD45RA, and CD62L (Supplementary Table S1). Accucheck counting beads (Life Technologies) were added to the cells for normalization. Next, the remaining CAR cells were pooled together and normalized based on expansion. CAR T cells were redistributed to wells, and fresh tumor cells were added to each well. The plates were incubated for 48 h at 37 °C and 5% CO_2_. Luminescence measurements and normalization were repeated until a loss of cytotoxicity was observed.

### Cystatin A enzyme-linked immunosorbent assay (ELISA)

The cystatin A concentration was assessed using a Human Cystatin A ELISA kit (Invitrogen, catalog no. EH140RB). Total cell lysates were extracted from CAR T cells using radioimmunoprecipitation assay (RIPA) buffer (Thermo) containing protease inhibitor cocktail (Roche). The total protein concentration was determined using a Pierce BCA assay (Thermo). Cystatin A levels were determined by ELISA according to the manufacturer’s instructions (Invitrogen) and calculated using standard curve interpolation. The absorbance was measured at the recommended wavelength on a Spark multimode plate reader (Tecan).

### CTSB activity assay

CTSB activity was assessed using the InnoZyme CTSB Activity Assay Kit (MilliPore Sigma, catalog no. CBA001). Total cell lysates were extracted from CAR T cells using the provided cell lysis buffer according to the manufacturer’s instructions. The total protein concentration was determined using a Pierce BCA assay (Thermo Fisher Scientific). CTSB activity was determined fluorometrically according to the manufacturer’s instructions (Calbiochem). Fluorescence was measured on a Spark multimode plate reader (Tecan).

### Treatment of CAR T cells with inhibitors

FMC63 CAR T cells were treated with small-molecule inhibitors targeting Actin (inhibitor: Cytochalasin D, Sigma Aldrich catalog no. C8273), Dynamin (inhibitor: DynaSore, Sigma Aldrich catalog no. D7693), CTSB (inhibitor: Ca-074-Me, SelleckChem catalog no. S7420), Clathrin (inhibitor: PitStop, Abcam catalog no. ab120687), or LFA-1 (inhibitor: BI-1950, Boehringer Ingelheim) at the indicated concentrations for 1 h. Following treatment, the cells were washed with T-cell media and centrifuged at 400×*g* for 5 min. To determine the effect of the nonmembrane-permeable CTSB inhibitor CA-074 (Millipore Sigma, catalog no. 205530), the inhibitor was added directly to the coculture at the indicated concentrations. As a control, CAR T cells were treated with DMSO. CAR T cells were cocultured with CD19-expressing tumor cells for the indicated intervals.

### Western blot

Total cell lysates were extracted from CAR T cells using RIPA buffer (Thermo Fisher Scientific) containing protease inhibitor (Roche). The total protein concentration was determined using a Pierce BCA assay (Thermo Fisher Scientific). Samples were separated by SDS–polyacrylamide gel electrophoresis, and separated proteins were transferred to nitrocellulose membranes using an iBlot2 transfer system (Thermo Fisher Scientific). Membranes were blocked with 5% nonfat milk–tris-buffered saline and incubated with primary antibodies against CD19, mCSTA, CISH, or β-actin. Membranes were washed and developed using species-specific secondary anti-IgG/horseradish peroxidase antibodies (R&D Systems) and Western Lightning Plus-ECL solution (PerkinElmer). Bands were visualized and quantified on an iBright 1500 imaging system (Thermo Fisher Scientific).

### In vivo cystatin A toxicity

Six- to eight-week-old NOD. *Cg-Rag1*^*tm1Mom*^*Il2rg*^*tm1Wjl*^/SzJ (NRG) mice (Jackson Laboratory) were irradiated with a sublethal dose of 200 cGy (Rad-Source 2000) and injected with the indicated number of CAR T cells into the lateral tail vein on the same day. To assess toxicity, animals were weighed daily and monitored for signs of distress in accordance with institutional regulations. After 14 days, the mice were euthanized, and the lungs, spleens, liver, and gastrointestinal organs were collected, sectioned, and subjected to H&E staining to assess organ structure. Slides were imaged using a BZ-X810 fluorescence microscope (Keyence) and a BZ-X800 analyzer.

### Secretome analysis

CAR and CAR_CSTA_ secretomes were analyzed by Codeplex assay (IsoPlexis). Following production and cryopreservation, CAR and CAR_CSTA_ T cells produced from three healthy donors were thawed and cultured in T-cell media + 40 IU IL-2 for 48 h. Cells were activated using anti-CD3/CD28 activation beads (Thermo Fisher) and incubated for 24 h. Supernatants were collected after 24 h, and cytokine profiling was measured using IsoPlexis Codeplex (Kcas Bio).

### Bulk RNA sequencing

Following production and cryopreservation, CAR and CAR_CSTA_ T cells from two independent productions were thawed and cultured in T-cell media + 40 IU/mL IL-2 for 48 h. After 48 h, CAR T cells were washed with FACS buffer, stained with anti-CD3, anti-HA, and DAPI (Supplementary Table S1), and sorted for CAR expression. Sorted CAR T cells were then pelleted and flash frozen. RNA was extracted, polyA-enriched, and sequenced using Illumina NovaSeq S4 PE100 sequencing targeting 10 million read pairs per sample.

### In vivo cystatin A model of CAR T-cell tumor control and persistence

Six- to eight-week-old male NOD. *Cg-Rag1*^*tm1Mom*^*Il2rg*^*tm1Wjl*^/SzJ (NRG) mice (Jackson Laboratory) were irradiated with a sublethal dose of 200 cGy (Day 0). The starting group size (*n* = 5) was based on a power calculation assuming a minimum effect size of 17%, a standard deviation/variance of 10%, and an alpha level of 0.05, according to the following formula:

$$n=\frac{({\sigma }_{1}^{2}+{\sigma }_{2}^{2}){(z1-\alpha /2+z1-\beta )}^{2}}{{\Delta }^{2}}$$

*n*: sample size; σ_1/2_: variance for each mean; *z*: critical *Z* value for a given α/β; α: probability of type I error; β: probability of type II error; ∆: difference between means.

After irradiation, mice were injected with 4 × 10^5^ NALM6 tumor cells via tail vein injection. On Day 4, mice were injected with 1 × 10^6^ FMC63 CAR or CAR_CSTA_ T cells or CAR T cells lacking a binding domain (∆scFv) via tail vein injection. Animals were weighed twice weekly and monitored for signs of distress in accordance with institutional regulations. Tumor burden was assessed weekly in the prone and supine positions by an in vivo imaging system (IVIS). For in vivo imaging, mice received an intraperitoneal injection of 3.3 mg D-luciferin (GOLDBIO # LUCK-10G). On day 28, animals were euthanized, and tissues were collected for analysis by flow cytometry. Average radiance values (p/s/cm²/sr) were determined using Living Image 4.8 software (PerkinElmer).

### In vivo model of CAR T-cell solid tumor infiltration

Six- to eight-week-old NOD. *Cg-Rag1*^*tm1Mom*^*Il2rg*^*tm1Wjl*^/SzJ (NRG) mice (Jackson Laboratory) were irradiated with a sublethal dose of 200 cGy (Rad-Source 2000) and injected intratibially on the same day with the indicated number of luciferase-expressing A673 cells (A673-Fluc) expressing CD19-GFP. The starting group size (*n* = 3) was based on a power calculation using the formula shown above, assuming a minimum effect size of 25%, a standard deviation of 10%, and an alpha level of 0.05. One animal was not available for analysis due to insufficient tumor growth. On day 14 after tumor cell injection, the indicated number of ∆scFv, FMC63 CISH^WT^ CAR, or CISH^KO^ CAR_CSTA_ T cells was injected into the lateral tail vein. Four days after CAR T-cell injection, mice were euthanized, and tumors were collected and dissociated using the Human Tumor Dissociation Kit (Miltenyi Biotec, cat # 130-095-929) and the OctoMACS tissue dissociator (Miltenyi Biotec). CAR T cells were quantified and phenotyped by flow cytometry.

### In vivo model of CAR T-cell solid tumor control

Six- to eight-week-old NOD. *Cg-Rag1*^*tm1Mom*^*Il2rg*^*tm1Wjl*^/SzJ (NRG) mice (Jackson Laboratory) were irradiated with a sublethal dose of 200 cGy (Rad-Source 2000) and injected intratibially on the same day with the indicated number of luciferase-expressing A673 cells (A673-Fluc) expressing CD19-GFP. The starting group sizes (*n* = 4–5) were based on a power calculation using the formula shown above, assuming a minimum effect size of 17-20% between the respective groups, a standard deviation of 10%, and an alpha level of 0.05. Four animals were not available for analysis due to unscheduled deaths. On day 7 after tumor cell injection, the indicated number of ∆scFv, FMC63 CISH^WT^ CAR, or CISH^KO^ CAR_CSTA_ T cells was injected into the lateral tail vein. Tumor burden was assessed weekly by an IVIS. For in vivo imaging, mice received an intraperitoneal injection of 3.3 mg D-luciferin (GOLDBIO # LUCK-10G). Peripheral blood was collected on day 19 after CAR T-cell injection for CAR T-cell quantification and phenotyping by flow cytometry.

### Statistical analysis

The respective statistical tests are stated in the figure legends. Generally, statistical significance between two groups was determined by a two-sided Student’s *t-*test or Mann‒Whitney *U*-test. Statistical significance between groups of three or more was determined by one- or two-way analysis of variance (ANOVA). All statistical tests were performed using Prism 10 (GraphPad). The results were considered significant when *p* < 0.05. ^*^ *p* < 0.05; ^**^ *p* < 0.01; ^***^p < 0.001; ^****^p < 0.0001.

## Supplementary information

Supplementary Material

## Data Availability

All data associated with this study are in the paper or supplementary materials. The CAR and CSTA constructs generated as part of this study are available under a Material Transfer Agreement from the corresponding author. Bulk RNA sequencing results are available at 10.5281/zenodo.18625446.
