# Supplementary material for: Preventing trogocytosis by cathepsin B inhibition augments CAR T-cell function
Source: Signal Transduct Target Ther. 2026 Apr 22;11:149. doi: 10.1038/s41392-026-02654-z (PMC13103101; doi:10.1038/s41392-026-02654-z)
Supplement: Supplementary file 1 — Supplementary Material [file 41392_2026_2654_MOESM1_ESM.docx]

**Supplementary Materials for**

*Preventing trogocytosis by cathepsin B inhibition augments CAR T cell function*

Kenneth A. Dietze, Kiet Nguyen, Aashli Pathni, Frank Fazekas, Wenxiang Sun,
Ethan Rosati, Jillian M. Baker, Maday Galeana Figueroa, Etse Gebru,
Daniel Yamoah, Rediet Mulatu, Alexander Wang, Aaron P. Rapoport,
David H. Lum, Xiaoxuan Fan, Sabarinath V. Radhakrishnan, Djordje Atanackovic,
Arpita Upadhyaya, Tim Luetkens

Correspondence to: [tluetkens@som.umaryland.edu](mailto:xxxxx@xxxx.xxx)

**This PDF file includes:**

Figures S1 to S8

Table S1

**SUPPLEMENTARY FIGURES**

**Supplementary Figure S1: CAR T cells acquire target antigen via trogocytosis.** **(a)** Schema of CAR-mediated trogocytosis (CMT). **(b)** Amounts of CD19 on CAR T cells lacking an antigen binding domain (∆scFv) or CD19 CAR T cells (FMC63) after a 1-hour coculture with CD19-positive cell lines at a 0.5:1 effector-target ratio as determined using flow cytometry. **(c)** Amounts of CD19 on tumor cells after a 1-hour coculture with ∆scFv or FMC63 CAR T cells at a 0.5:1 effector-target ratio as determined using flow cytometry. **(b/c)** Data is representative of at least three independent experiments. **(d)** Confocal imaging z-stack showing antigen transfer from CD19-GFP-expressing 293T cells to FMC63 CAR T cells after 10 minutes. CAR is shown in cyan, CD19 is shown in green, and cathepsin B is shown in red. Scale bar represents 5µm. **(e)** LINGO1 transfer to ∆scFv CAR T cells or CAR T cells targeting LINGO1 after a 1-hour coculture with the Ewing sarcoma cell line A673 at a 1:1 effector-target ratio as determined by flow cytometry. **(f)** GD2 transfer to ∆scFv CAR T cells or CAR T cells targeting GD2 after a 1-hour coculture with the GD2+ Ewing sarcoma cell line TC-71 at a 1:1 effector-target ratio using flow cytometry. **(e/f)** Data is representative of two independent experiments. **(g)** Folate receptor alpha (FolR⍺) transfer to CAR T cells targeting FolR⍺ or an irrelevant CAR after a 1-hour coculture with the FolR⍺+ ovarian cancer cell line SKOV3 at a 1:1 effector-target ratio using flow cytometry. **(h)** Gating strategy for assessment of trogocytosis in clinical samples. **(i)** Amounts of TIM-3 and PD-1 on CD19^-^ and CD19^+^ CAR T cells from the peripheral blood of a patient receiving CD19 CAR T cell therapy, as determined by flow cytometry.

**Supplementary Figure S2: CAR-mediated trogocytosis directly causes CAR T cell dysfunction.** **(a)** Western blot of lysates from 293 cells transduced with a CD19-GFP expression construct and subsequently transfected with TAD expression constructs. Blot is representative of two independent experiments. **(b)** Quantification of western blot in Suppl. Fig. S2A. Values are normalized to β-actin. **(c)** Fold change expansion of FMC63 CAR T cells ± TAD_GFP_/TAD_CD19_ during manufacturing as determined by cell counting, normalized to ∆scFv. Data represents mean ± S.D. from three independent CAR T cell productions using cells from 3 different healthy donors for FMC63 CAR T cells ± TAD_GFP_ and a single production for FMC63 TAD_CD19_ CAR T cells. **(d)** Survival of CD19-GFP-expressing NALM6-luc cells after 16-hour coculture with FMC63 CAR T cells ± TAD_GFP_/TAD_CD19_ or ∆scFv CAR T cells at the indicated effector-target ratios. Tumor survival was measured using a luciferase-based cytotoxicity assay. Data represent mean ± S.D. of three technical replicates and is representative of three independent experiments. **(e)** Percentage of CAR T cells positive for CD19 following a 1-hour coculture with CD19-GFP-expressing NALM6 cells as determined by flow cytometry. **(f)** Percentage of CAR T cells positive for GFP following a 1-hour coculture with CD19-GFP-expressing NALM6 cells as determined by flow cytometry. **(e/f)** Data is representative of three independent experiments. **(g)** Mean fluorescence intensity of Ki67 in CAR T cells after a 24-hour coculture with CD19-GFP-expressing NALM6 cells as determined by flow cytometry. Data is representative of two independent experiments. **(h)** Percentage of CAR T cells positive for LAG-3 after a 24-hour coculture with CD19-GFP-expressing NALM6 cells using full-spectrum flow cytometry. **(i)** Percentage of CAR T cells positive for TIM-3 after a 24-hour coculture with CD19-GFP-expressing NALM6 cells using full-spectrum flow cytometry. **(h/i)** Data is representative of three independent experiments. **(e-i)** Data represent mean ± S.D. of 3 technical replicates. Statistical significance was determined by one-way ANOVA.

**Supplementary Figure S3: Validation of luciferase complementation assay. (a)** Luminescence of 293T cells 24 hours after transfection with nLuc, cLuc, or nLuc+cLuc containing expression constructs. Statistical significance was determined by one-way ANOVA. **(a)** Luminescence of nLuc+ or nLuc+cLuc+ CAR T cells after manufacturing. Statistical significance was determined by two-tailed Student’s *t*-test. **(a/b)** Data represent mean ± S.D. of 3 technical replicates. Data is representative of two independent experiments.

**Supplementary Figure S4: Inhibition of cathepsin B reduces trogocytosis in a Ewing sarcoma model.** Membrane transfer to CAR T cells following a 3-hour coculture of ∆scFv or LINGO1-targeting CAR T cells and the Ewing sarcoma cell line A673 at a 1:1 effector-target ratio was determined by flow cytometry. CAR T cells were pre-treated with DMSO or 100µM Ca-074-Me for 1 hour prior to coculture. Data represent mean ± S.D. of 3 technical replicates.

**Supplementary Figure S5: Overexpression of CSTA does not alter CAR T cell expansion, phenotype, or cytokine secretion. (a)** Expansion of ∆scFv, FMC63 CAR or FMC63 CAR_CSTA_ T cells during manufacturing as determined by cell counting. **(b)** Expansion of ∆scFv, CL10 CAR, or CL10 CAR_CSTA_ CAR T cells during manufacturing as determined by cell counting. **(c)** FMC63 or **(d)** CL10 CAR or CAR_CSTA_ T cells were analyzed by flow cytometry after production for *I:* CAR expression, *II*: T cell subsets, and *III-IV:* T cell phenotype. **(e)** Comparison of secretome between FMC63 CAR and CAR_CSTA_ T cells after 24 hours of activation using anti-CD3/CD28 beads as measured by CodePlex assay. Data represents the average of CAR T cell products manufactured from three healthy donors .

**
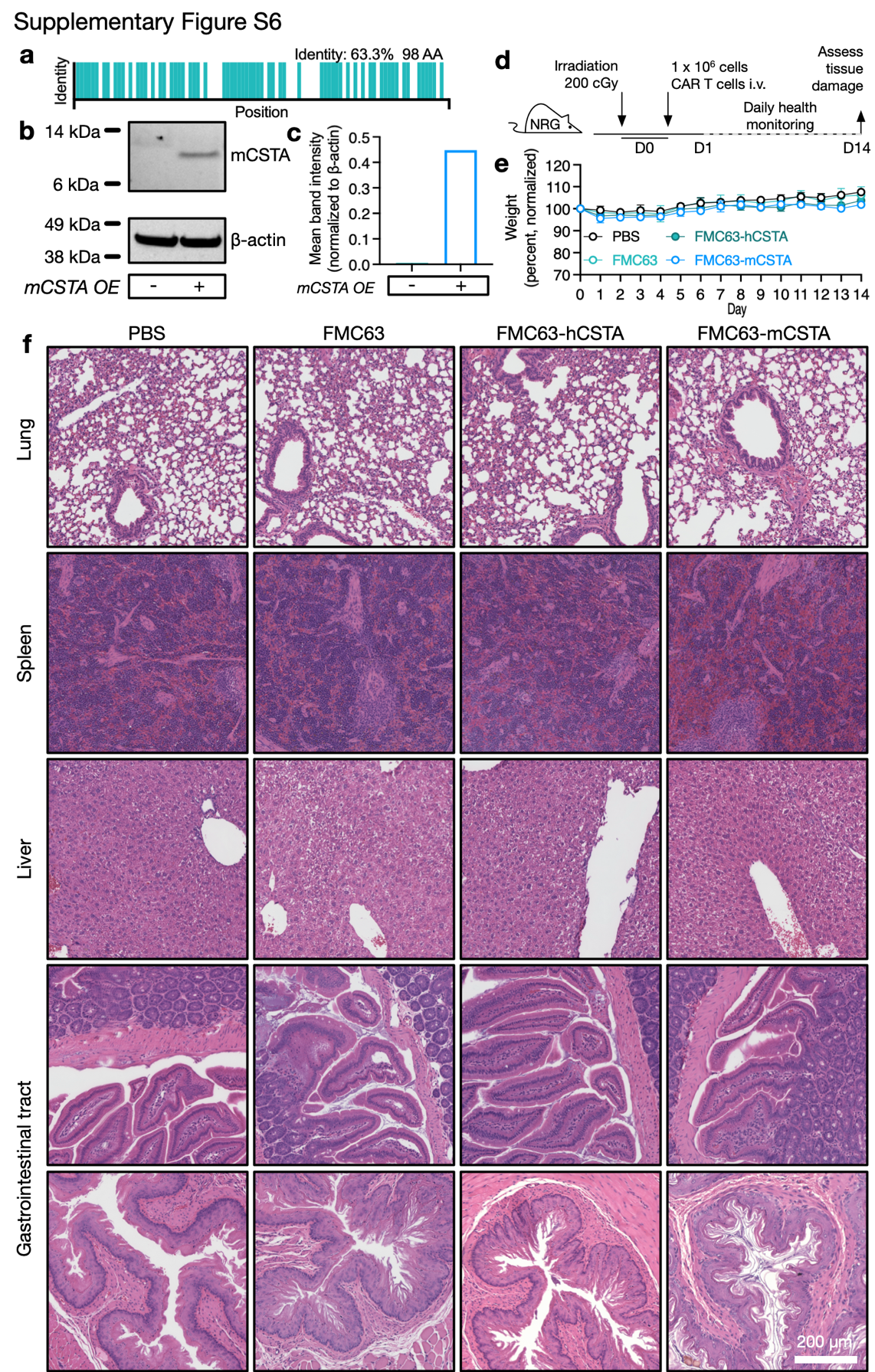
**

**Supplementary Figure S6: Overexpression of CSTA in T cells does not cause toxicity in mice. (a)** Homology between human cystatin A (hCSTA, Uniprot P01040) and mouse cystatin A (mCSTA, Uniprot P56567) as determined by blastp. **(b)** Expression of mCSTA in lysates from human FMC63 CAR T cells engineered to overexpress mCSTA as determined by western blot. **(c)** Quantification of western blot in Suppl. Fig. S6B. **(d)** Schema of *in vivo* experiment to assess potential toxicity of hCSTA or mCSTA overexpression. **(e)** Weights of mice treated with PBS, conventional FMC63, or FMC63 CAR T cells engineered to overexpress hCSTA or mCSTA. Data represent mean ± S.D. of 3 mice per group. **(f)** H&E staining of lungs, spleens, livers, and gastrointestinal tracts of mice treated with PBS, conventional FMC63 CAR T cells, or FMC63 CAR T cells overexpressing human CSTA or mouse CSTA. Images are representative of samples from three mice per group.

**Supplementary Figure S7: Cystatin abundance regulates CMT in vitro and in vivo without reducing short-term cytotoxicity. (a)** Survival of NALM6-, Daudi-, and Toledo-luc cells after a 16-hour coculture with FMC63 CAR or CAR_CSTA_ T cells at the indicated effector-target ratios. Data represent mean ± S.D. of three technical replicates. Data is representative of at least three independent experiments using cells produced from three healthy donors. **(b)** Survival of MM.1S-, RPMI8226-, and U266B1-luc cells after 16-hour coculture with CL10 CAR or CAR_CSTA_ T cells at the indicated effector-target ratios. Data represent mean ± S.D. of three technical replicates and is representative of three independent experiments. **(a/b)** Tumor survival was measured using a luciferase-based cytotoxicity assay. **(c)** Area-under-curve quantification of CMT as determined by CompLuc assay in Fig. 5F. **(d)** Area-under-curve quantification of CMT as determined by CompLuc assay in Fig. 5G. **(c/d)** Data represent mean ± S.D. of three technical replicates. Statistical significance was measured using a two-tailed Student’s *t*-test. **(e)** Mean fluorescence intensity of CD69 in FMC63 CAR or CAR_CSTA_ T cells using flow cytometry. Statistical significance was measured using a two-tailed Student’s *t*-test. Data represent mean ± S.D. of 3 technical replicates. Data is representative of two independent experiments. **(f)** Schema of interaction between full-length CSTA or CSTA_1-57_ with CTSB. **(g)** Homology between human cystatin A (Uniprot P01040) and cystatin B (Uniprot P04080) as determined by blastp. **(h)** CMT as determined by CompLuc during co-culture of K562 cells expressing CD19-cLuc and FMC63 CAR T cells ± CSTA_1-57_/CSTB. Data represents best fit of three technical replicates and is representative of at least three independent experiments. **(i)** Area-under-curve quantification of luminescence in Suppl. Fig. S7H. Data represent mean ± S.D. of three technical replicates. **(j)** Percentage of CAR T cells positive for CD19 following a 30-minute coculture of FMC63 CAR or CAR_CSTA_ T cells and K562 cells expressing CD19-cLuc at a 0.5:1 effector-target ratio as determined by flow cytometry. Data represent mean ± S.D. of 3 technical replicates. Statistical significance was determined using one-way ANOVA. **(k)** Histograms depicting CD19 transfer to CAR T cells following a 30-minute coculture of FMC63 CAR or CAR_CSTA_ T cells and K562 cells expressing CD19-cLuc at a 0.5:1 effector-target ratio as determined by flow cytometry. **(j/k)** Data is representative of at least three independent experiments using cells produced from three healthy donors. **(l)** Percentage of CAR T cells positive for GFP following a 1-hour coculture of FMC63 CAR or CAR_CSTA_ T cells and A673 cells expressing CD19-GFP at a 1:1 effector-target ratio as determined by flow cytometry. Data is representative of two independent experiments. **(m)** CD19 transfer to CAR T cells following a 30-minute coculture of FMC63 CAR, CAR_CSTA 1-57_, or CAR_CSTB_ T cells and K562 cells expressing CD19-cLuc at a 0.5:1 effector-target ratio. **(n)** Amount of CD19 on tumor cells following a 30-minute coculture of FMC63 CAR, CAR_CSTA 1-57_, or CAR_CSTB_ T cells and K562 cells expressing CD19-cLuc at a 0.5:1 effector-target ratio. **(o)** Total CAR T cells following a 30-minute coculture of FMC63 CAR, CAR_CSTA 1-57_, or CAR_CSTB_ T cells and K562 cells expressing CD19-cLuc at a 0.5:1 effector-target ratio. CAR T cell numbers are normalized to wells containing only CAR T cells using counting beads. Data represent mean ± S.D. of three technical replicates. **(p)** Number of B-ALL cells following a 1-hour coculture of FMC63 CAR or CAR_CSTA_ T cells and primary B-ALL cells at a 1:1 effector-target ratio as determined by flow cytometry. Circles indicate three technical replicates from a single patient B-ALL sample. **(l-p)** Data represent mean ± S.D. of 3 technical replicates and statistical significance was determined using one-way ANOVA. **(l-o)** Data is representative of at least three independent experiments.

**Supplementary Figure S8: CAR_CSTA_ T cells retain benefits of reduced trogocytosis following CISH knockout. (a)** Tumor burden in 5 mice per group bearing systemic NALM6 tumors and treated with intravenous ∆scFv, FMC63 CAR, or FMC63 CAR_CSTA_ T cells as determined by IVIS. Data is representative of two independent experiments. **(b)** Amounts of TIM-3 in FMC63 CAR or CAR_CSTA_ T cells throughout an *in vitro* serial coculture with CD19-GFP-expressing NALM6-luc cells as determined by flow cytometry. Statistical significance was determined using multiple two-tailed Student’s *t*-tests. Data represent mean ± S.D. of 3 technical replicates. Data is representative of two independent experiments. **(c)** Western blot showing expression of CISH in CAR_CSTA_ CISH^WT^ and CISH^KO^ T cells at the end of manufacturing. **(d)** Quantification of western blot in Suppl. Fig. S8C. Values are normalized to β-actin. **(e)** Phenotype of FMC63 CAR_CSTA_ CISH^WT^ or CISH^KO^ CAR T cells following production as determined by flow cytometry. **(f)** Amount of CD19 on CAR T cells following a 1-hour coculture of FMC63 CAR, CAR_CSTA_ CISH^WT^, or CAR_CSTA_ CISH^KO^ T cells and NALM6 cells expressing CD19-GFP at a 1:1 effector-target ratio as determined by flow cytometry. **(g)** Amount of CD19 on tumor cells following a 1-hour coculture of FMC63 CAR, CAR_CSTA_ CISH^WT^, or CAR_CSTA_ CISH^KO^ T cells and NALM6 cells expressing CD19-GFP at a 1:1 effector-target ratio as determined by flow cytometry. **(h)** Number of CAR T cells following a 1-hour coculture of FMC63 CAR, CAR_CSTA_ CISH^WT^, or CAR_CSTA_ CISH^KO^ T cells and NALM6 cells expressing CD19-GFP at a 1:1 effector-target ratio as determined by flow cytometry. CAR T cell numbers are normalized to wells containing only CAR T cells using counting beads. **(f-h)** Statistical significance was determined using one-way ANOVA. Data represent mean ± S.D. of 3 technical replicates. Data is representative of three independent experiments. **(i)** Tumor burden in 5 mice per group bearing systemic NALM6 tumors and treated with intravenous ∆scFv, FMC63 CAR, or FMC63 CAR_CSTA_ CISH^WT^ or CISH^KO^ T cells as determined by IVIS. **(j)** Tumor burden in 3-4 mice per group bearing intratibial A673 tumors and treated with intravenous ∆scFv, FMC63 CAR, or FMC63 CAR_CSTA_ CISH^KO^ T cells as determined by IVIS. **(k)** Phenotype of intratumoral CAR T cells from mice bearing intratibial A673 tumor cells four days after CAR T cell injection as determined by flow cytometry. Data represent mean ± S.D. from 2-3 animals per group. Statistical significance was determined by two-way ANOVA.

**SUPPLEMENTARY TABLES**

| **Target** | **Flurophore** | **Clone** | **Supplier** | **Target cells** |
| --- | --- | --- | --- | --- |
| BCMA | PE, APC | 19F2 | Biolegend | Multiple |
| CAR detection reagent (FMC63) | PE | n/a | Miltenyi | CAR T cells |
| Cathepsin B | Biotin | n/a | R&D Systems | Western blot |
| Cathepsin L | Biotin | n/a | R&D Systems | Western blot |
| CD3 | APC, FITC, PE, PE-Cy7, PerCP, BV421, BV650 | UCHT1 | Biolegend | Pan-T |
| CD4 | BUV496 | SK3 | BD Biosciences | CD4 T cells |
| CD8 | BUV563, FITC, PE | RPA-T8 | BD Biosciences | CD8 T cells |
| CD19ext | APC, BV650, BV711, PE, FITC, biotin | HIB19 | Biolegend | Multiple |
| CD19int | PE | D4V4B | Cell signaling Technology | Multiple |
| CD20 | APC, BV421, PE-Cy7 | 2H7 | Biolegend | Tumor |
| CD45 | APC | 2D1 | Biolegend | T cells |
| CD45RA | PE/Cy7 | HI100 | Biolegend | T cell subsets |
| CD62L | BUV395, BV421 | SK11 | BD Biosciences | T cell subsets |
| CD69 | PE | FN50 | Biolegend | T cell subsets |
| CD95 | BV510 | DX2 | Biolegend | T cell subsets |
| Cell Trace Far Red | n/a | n/a | Invitrogen | T cells |
| Cytokine-inducible SH2-containing protein | n/a | n/a | Cell signaling Technology | Western blot |
| DAPI | n/a | D1306 | Life Technologies | Live/Dead |
| FolR⍺ | PE | LK26 | Biolegend | Multiple |
| GD2 | APC | 14G2A | Biolegend | Multiple |
| Human Cystatin A | Biotin | n/a | Sino Biological | Western blot |
| Human Cystatin B | n/a | n/a | Sino Biological | Western blot |
| Hemagglutinin | PE, PE-Cy7, APC | 16B12 | Biolegend | CAR T cells |
| HER2t | Alexa Fluor 647 | Hu5 | R&D Systems | CAR T cells |
| Ki67 | PE | Ki-67 | Biolegend | T cell subsets |
| LAG-3 | Alexa Fluor 700 | 11C3C65 | Biolegend | T cell subsets |
| LINGO1 | Biotin | Opicinumab | Medchemexpress | Multiple |
| Murine Cystatin A | n/a | Polyclonal | Proteintech | Western blot |
| PD-1 | APC-Cy7, PE, PE-Cy7 | EH12.2H7 | Biolegend | T cell subsets |
| TIM-3 | BV421 | 7D3 | BD Biosciences | T cell subsets |
| Zombie Aqua | n/a | n/a | Biolegend | Live/Dead |
| Zombie Red | n/a | n/a | Biolegend | Live/Dead |
| Zombie Violet | n/a | n/a | Biolegend | Live/Dead |
| Zombie NIR | n/a | n/a | Biolegend | Live/Dead |
| Streptavidin | Alexa Fluor 647 | n/a | Jackson Immunoresearch | Multiple |
| β-actin | Unconjugated | 937215 | R&D Systems | Western blot |
| Plasma membrane | Biotracker 555 | n/a | Millipore Sigma | Multiple |

**Supplementary Table S1: Table of monoclonal antibodies and viability dyes used for flow cytometry and western blot analyses.**
